# Temperature-regulated heterogeneous extracellular matrix gene expression defines biofilm morphology in *Clostridium perfringens*

**DOI:** 10.1038/s41522-020-00139-7

**Published:** 2020-07-31

**Authors:** Nozomu Obana, Kouji Nakamura, Nobuhiko Nomura

**Affiliations:** 1 Transborder Medical Research Center, Faculty of Medicine, University of Tsukuba, 1-1-1 Tennodai, Tsukuba, Ibaraki 305-8577 Japan; 2 Microbiology Research Center for Sustainability, University of Tsukuba, 1-1-1 Tennodai, Tsukuba, Ibaraki 305-8572 Japan; 3 Faculty of Life and Environmental Sciences, University of Tsukuba, 1-1-1 Tennodai, Tsukuba, Ibaraki 305-8572 Japan

**Keywords:** Biofilms, Microbial genetics

## Abstract

Cells in biofilms dynamically adapt to surrounding environmental conditions, which alters biofilm architecture. The obligate anaerobic pathogen *Clostridium perfringens* shows different biofilm structures in different temperatures. Here we find that the temperature-regulated production of extracellular polymeric substance (EPS) is necessary for morphological changes in biofilms. We identify BsaA proteins as an EPS matrix necessary for pellicle biofilm formation at lower temperature and find that extracellularly secreted BsaA protein forms filamentous polymers. We show that *sipW-bsaA* operon expression is bimodal, and the EPS-producing population size is increased at a lower temperature. This heterogeneous expression of the EPS gene requires a two-component system. We find that EPS-producing cells cover EPS-nonproducing cells attaching to the bottom surface. In the deletion mutant of *pilA2*, encoding a type IV pilin, the EPS gene expression is ON in the whole population. This heterogeneity is further regulated by the cleavage of the *pilA2* mRNA by RNase Y, causing temperature-responsive EPS expression in biofilms. As temperature is an environmental cue, *C. perfringens* may modulate EPS expression to induce morphological changes in biofilm structure as a strategy for adapting to interhost and external environments.

## Introduction

Pathogenic bacteria adapt to both the host-internal and external environments for infection and survival. They must be able to recognize these different environments, which leads to a differential response to be tolerant to various stresses in each environment. Strictly anaerobic pathogens, as well as facultative anaerobes, are widespread throughout the environment, with a habitat inside the host in areas such as the gastrointestinal tract. Oxygen and desiccation are environmental stresses that must be avoided in the external environment for strictly anaerobic pathogens to be able to be transmitted to different hosts. Determining how anaerobic pathogens respond to environmental signals outside the host and which biological processes are involved in adaptation to the environment are crucial for understanding the pathogenesis of anaerobic bacteria.

To overcome environmental stresses, one survival strategy is biofilm formation. Most bacteria natively form biofilms, which are microbial multicellular communities. Cells in biofilms are surrounded by a self-produced matrix, known as extracellular polymeric substances (EPS), which are mainly composed of extracellular nucleic acids, proteins, and polysaccharides, although the specific composition varies across species^1^. The higher-order structure of biofilms is supported by EPS, which simultaneously confers tolerance to desiccation, oxidative stresses, and external antimicrobials^2^. Thus, biofilm formation is thought to be a crucial ability for anaerobic pathogens to survive various internal/external environments. Thus, to understand biofilm properties and to develop antibacterial strategies, identification of the specific composition of EPS in biofilms associated with the biofilm architecture is crucial.

*Clostridium perfringens* is a gram-positive spore-forming bacterium that is a causative agent of food poisoning, gas gangrene, and antibiotic-associated diarrhea because it produces numerous toxins and extracellular enzymes^3^. This bacterium is an obligate anaerobe but is found widely in environments such as soil and the intestines of animals due to its spore-forming ability. Recently, *C. perfringens* was found to form biofilms, which provide increased tolerance to antibiotics and oxidative stresses^4,5^. Sporulation provides extreme resistance to environmental stresses, but spores are highly dormant and cannot quickly respond to environmental changes. Therefore, in the natural environment, both sporulation and biofilm formation are survival strategies for *C. perfringens* and are thus related to its pathogenesis. The EPS of *C. perfringens* biofilms is reported to consist of extracellular DNA, extracellular proteins, and polysaccharides^5–8^. Type IV pili are extracellular appendages involved in attachment to host cells and are described as a component of the biofilm necessary for maximal biofilm formation^5,9,10^. However, the other genes involved in EPS production in *C. perfringens* are not well known.

Extracellular protein has been demonstrated to be a major component of many bacterial biofilms. In the gram-positive sporulating bacterium *Bacillus subtilis*, the TasA and BslA proteins have been reported to be biofilm EPSs, and their production is required for the formation of air–liquid or solid–air interface biofilms by these organisms^11–13^. In *Staphylococcus aureus*, Bap protein is localized at the cell surface, which facilitates cell attachment to the substrate and cell-to-cell interactions^14^. These biofilm EPS genes are typically regulated in response to environmental signals. Furthermore, several genes involved in biofilm formation, such as *tasA*, show heterogeneous expression within a population^15^. In addition, biofilm cells often display localized gene expression, which leads to phenotypic heterogeneity and multicellular behaviors^16,17^. The localized gene expression in the biofilm formed by strictly anaerobic pathogens has been poorly investigated, so far, due to the limitation of the methods to visualize and analyze the gene expression at the single-cell level in the anaerobic condition.

In a recent study, we showed that temperature drastically influences *C. perfringens* biofilm morphology, which indicates that *C. perfringens* modulates gene expression involved in biofilm formation in response to external environmental temperature^7^. At “higher” temperatures (37 °C), *C. perfringens* attach to surfaces, where the cells pack densely in a biofilm. Hereafter we refer to this structure an adherent biofilm. In contrast, at ambient temperature (25 °C), the attachment activity to the surface is decreased, and elastic thick pellicle-like biofilms are built. This pellicle-like biofilm, hereafter referred to as the pellicle biofilm, is located near the bottom surface, but the pellicle biofilm does not strongly attach to the surface. Virulence factor production and biofilm formation of pathogenic bacteria are frequently regulated by temperature^18–28^. Temperature is recognized as a signal involved in pathogenesis since the temperature inside the host is usually higher than the temperature of the outside environments^29,30^. Therefore, morphological changes in the biofilm could be an adaptive strategy of this bacterium. In addition, we found that at a lower temperature, *C. perfringens* produces filamentous EPS, implying that temperature-regulated EPS production facilitates morphological changes in the biofilm.

Biofilms contain heterogeneous populations, which leads to multicellular behaviors. Differentiation into specialized cell types in the bacterial population permit the preparation for quick and drastic change^31^. The emergence of specialized cells is essential for the development of biofilm architectures^32^. However, evidence for the spatiotemporal differentiation of gene expression in biofilms is limited to several model anaerobes. In the present study, we identified the CPE0515 gene as responsible for EPS production, which leads to morphological changes in the biofilms of *C. perfringens*. We found that extracellular proteins assemble to form the filamentous structures that cover the biofilm surface. Thus, we named the EPS gene *bsaA* for biofilm self-assembling protein. We used anaerobic fluorescent protein reporters to visualize the localized gene expression within the anaerobic biofilms. These data imply that the localized heterogeneous gene expression involved in EPS production within the biofilm allows the division of labor and environmental adaptation in the anaerobic pathogen *C. perfringens*.

## Results

### Identification of genes responsible for pellicle biofilm formation

**Fig. 1:**
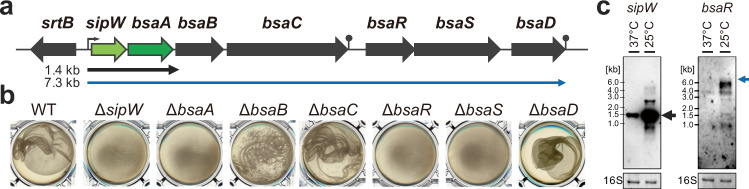
Identification of genes involved in pellicle biofilm formation. **a** Schematics of the *sipW-bsaABCRSD* operon. A bent arrow indicates the transcriptional start site. Predicted transcriptional terminators are shown. **b** Pellicle biofilm formation of the mutant strains. Cells were grown for 2 days at 25 °C. The edges of pellicle biofilms, formed at the bottom of the well, were picked by pipetting and photographed after gentle agitation. **c** Northern blot analysis using *sipW* and *bsaR* gene-specific DNA probes. WT *C. perfringens* cells were grown to the mid-exponential phase (OD_600_ = 1.0) at 37 or 25 °C. In each lane, 1 μg of total RNA was loaded. 16S rRNAs stained with methylene blue are shown at the bottom as a loading control.

Previously, we reported that morphological changes in biofilm structure depend on the growth temperature of *C. perfringens*^7^. Cells attach to the bottom surface of the microtiter plate well and form thin biofilms at 37 °C. In contrast, when grown at 25 °C, cells produce filamentous EPS and form thick elastic pellicle-like biofilms near the bottom of the well, but most cells are not attached to the surface. Thus, *C. perfringens* produces biofilms with different structures at different growing temperatures. We refer to the biofilms formed at 37 °C as “adherent biofilms” and those formed at 25 °C as “pellicle biofilms”. To identify the genes responsible for pellicle biofilm formation of *C. perfringens* at 25 °C, we constructed a transposon mutant library of *C. perfringens* strain 13 and screened the library in 24-well microtiter plates to test the ability to form elastic pellicle biofilms. Of 1360 random transposon mutants, 51 strains were unable to form elastic pellicle biofilms (Supplementary Table 1). Among the genes with an inserted transposon, we focused on *bsaA*, the only gene encoding a protein with a signal sequence and predicted to be secreted into the extracellular milieu. Previously, we found that the *bsaA* gene is a part of the *sipW-bsaABCRSD* operon (Fig. 1a)^33^. The *sipW* encodes a putative signal peptidase, and *bsaS* and *bsaR* encode a sensor kinase and response regulator, respectively. The *bsaB*, *bsaC*, and *bsaD* encode putative secreted proteins. We constructed deletion mutants of these genes and tested their ability to form pellicle biofilms (Fig. 1b). We note that we used the HN13 strain for mutant construction, which is a Δ*galKT* mutant derivative of strain 13. We have previously shown that there is no obvious difference in pellicle biofilm formation between strain 13 and HN13^7^. Thus, hereafter, we refer to HN13 as wild type (WT) in this study. The deletion mutants of *sipW, bsaA, bsaR*, and *bsaS* did not produce pellicle biofilms but formed adherent biofilms (Fig. 1b). The *bsaB* and *bsaC* mutants showed fragile pellicle biofilm formation (Fig. 1b). Therefore, the *sipW, bsaA, bsaR*, and *bsaS* genes were necessary for pellicle biofilm formation, while the *bsaB* and *bsaC* genes improved it.

We reported that a temperature lower than 37 °C could induce pellicle biofilm formation, which suggests that temperature controls the expression of genes involved in pellicle biofilm formation. We isolated the total RNA from cells grown to the mid-exponential phase at 25 or 37 °C and compared the gene expression levels of the *sipW-bsaABCRSD* operon. We detected *sipW-bsaA* bicistronic mRNA (1.4 kb) and *sipW-bsaABCRSD* polycistronic mRNA (7.3 kb). Both transcripts showed higher levels of accumulation at 25 °C compared with 37 °C (Fig. 1c). We predicted a transcriptional terminator in the 3′ flanking region of *bsaA* and identified a transcriptional start site upstream of *sipW* (Supplementary Fig. 1a–c). Thus, both *sipW-bsaA* bicistronic mRNA and *sipW-bsaABCRSD* polycistronic mRNA were transcribed from the *sipW* promoter, where the latter was a readthrough product. We did not detect both mRNAs in the *bsaR* and *bsaS* mutants, indicating that the BsaR/BsaS two-component system was indispensable for the *sipW* operon expression (Supplementary Fig. 2a). These results indicated that lower temperatures induced expression of the *sipW-bsaA* operon necessary for pellicle biofilm formation.

### Extracellularly secreted BsaA protein forms HMW polymers

**Fig. 2:**
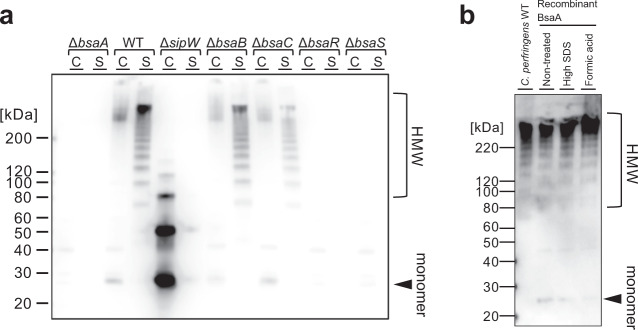
Polymerization of extracellularly secreted BsaA protein. **a** Western blotting of BsaA. Cell extracts (C) and culture supernatants (S) were isolated from WT or mutant strains grown to the mid-exponential phase (OD_600_ = 1.0) at 25 °C. Protein samples (OD_600_ unit = 0.01) were separated on a 4–12% gradient SDS-polyacrylamide gel. BsaA proteins were detected with anti-BsaA antisera. **b** Recombinant BsaA proteins also form polymers. Recombinant BsaA-6×His proteins were purified by affinity binding to Ni-NTA affinity resin from *E. coli*. BsaA-6×His proteins were incubated in 10% SDS for 10 min at 95 °C or in 20% formic acid for 10 min at the room temperature. Protein samples of *C. perfringens* WT cells (OD_600_ unit = 0.004) and purified BsaA-6×His proteins (10 ng) were separated on a 4–12% gradient SDS-polyacrylamide gel. BsaA proteins were detected by western blotting with anti-BsaA antisera. HMW protein polymers and protein monomers are shown.

The amino acid sequence of the BsaA protein contains a signal peptide, which suggests that BsaA is an extracellular protein. We determined the localization of the BsaA protein in biofilm cells by western blotting (Fig. 2a). Based on the amino acid sequence, the molecular weight of the BsaA protein was predicted to be ≈23 kDa. We detected a monomer of BsaA protein as well as high-molecular-weight (HMW) ladder patterns in the extracellular fraction (Fig. 2a). The results suggested that BsaA proteins formed stable polymers in the extracellular milieu. We also detected HMW smear bands in the whole cell fraction, which implied that some amount of extracellular HMW polymers was associated with cells. We did not detect these proteins in the *bsaA*, *bsaR*, and *bsaS* mutants (Fig. 2a). In the *sipW* mutant, extracellular BsaA proteins were not detected (Fig. 2a). In *bsaB* and *bsaC* mutants, compared with WT, the amount of extracellular BsaA HMW polymers was lowered (Fig. 2a). These results are consistent with pellicle biofilm formation in the mutant strains (Fig. 1b). The complementation of the plasmid expressing BsaA proteins restored the expression of extracellular HMW proteins and the formation of pellicle biofilms in the *bsaA* mutant (Supplementary Fig. 3). In addition, anti-BsaA antisera inhibited pellicle biofilm formation in a concentration-dependent manner (Supplementary Fig. 4). These results indicated that extracellular HMW BsaA protein was required for pellicle biofilm formation.

Certain gram-positive bacterial pilus components are covalently attached to each other and are not dissociated by SDS-PAGE; in these cases, proteins assembled in the pili show a HMW ladder pattern similar to the BsaA protein pattern^34^. Sortase is responsible for the polymerization of pilus subunits in several gram-positive bacteria^35^. The 5′ flanking region of the *sipW* gene contains a putative sortase-encoding gene (*srtB*) (Fig. 1a). We constructed a *srtB* deletion mutant and used it for the biofilm formation assay and western blotting of the BsaA protein (Supplementary Fig. 5). We observed no differences in either pellicle biofilm formation or BsaA protein profiles between the WT and *srtB* mutant. These results indicated that SrtB was apparently not involved in BsaA polymerization and pellicle biofilm formation.

To assess whether the HMW protein polymer of BsaA consisted solely of BsaA proteins, we expressed His-tagged BsaA proteins from which the signal peptide sequence was eliminated in *Escherichia coli*. The purified His-tagged protein from this strain also showed a HMW protein pattern comparable with BsaA protein expressed in *C. perfringens* (Fig. 2b). In addition, to assess the stability of BsaA polymers, we exposed the purified recombinant His-tagged BsaA proteins to a high-concentration SDS solution (350 mM) or 20% (v/v) formic acid, which are sufficiently harsh treatments that are able to disrupt stable structures such as amyloid fibers. After these treatments and subsequent boiling, BsaA proteins still formed HMW polymers comparable with non-treated BsaA proteins (Fig. 2b). This finding suggested that the HMW polymers, which were solely composed of BsaA proteins, were extremely stable.

### SipW, a putative signal peptidase, is necessary for extracellular localization of BsaA protein

**Fig. 3:**
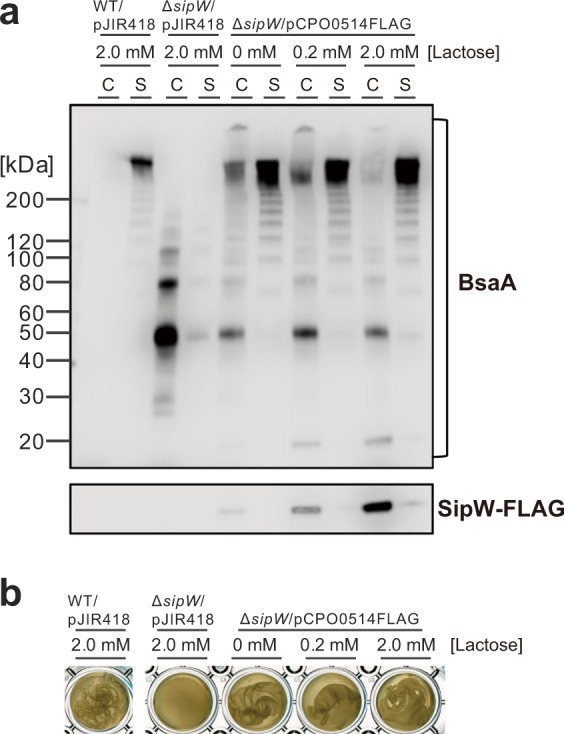
SipW signal peptidase is necessary for the secretion and polymerization of BsaA proteins. **a** Western blotting of BsaA proteins. Cell extracts (C) and culture supernatants (S) were isolated from WT or *sipW* mutant cells harboring the indicated plasmids. Protein samples (OD_600_ = 0.01) were separated on a 4–12% gradient SDS-polyacrylamide gel. BsaA and SipW-FLAG proteins were detected with anti-BsaA and anti-FLAG antisera, respectively. **b** The SipW-FLAG-expressing plasmid restores pellicle biofilm formation of the *sipW* mutant. Cells were grown for 2 days at 25 °C. The edges of pellicle biofilms, formed at the bottom of the well, were picked by pipetting and photographed after gentle agitation.

The *sipW* gene, the first gene in the *sipW-bsaABCRSD* operon, encodes a signal peptidase that cleaves signal peptides and is required for protein secretion. In *sipW* mutant cells, which did not produce pellicle biofilms, BsaA proteins were not detected in the extracellular fraction (Figs. 1b and 2a). In contrast, BsaA protein monomers (~23 kDa), dimers (~50 kDa), and trimers (~80 kDa) accumulated in the whole cell fraction (Fig. 2a). We constructed a SipW-expressing plasmid in which the expression of the FLAG-tagged *sipW* gene was controlled by the lactose-inducible *bgaL* promoter. We monitored the expression of SipW-FLAG protein by western blotting, and SipW-FLAG expression depended on the concentration of lactose (Fig. 3a). However, we also detected the SipW-FLAG protein under conditions without lactose, which might be caused by the leaky expression of the *bgaL* promoter (Fig. 3a). The complementation of the SipW-expressing plasmid restored the extracellular localization of BsaA proteins and pellicle biofilm formation in the *sipW* mutant (Fig. 3a, b). These results indicated that both BsaA polymerization into HMW proteins and their extracellular localization depended on SipW protein.

### Overexpression of SipW and BsaA from strain 13 allows pellicle biofilm formation in SM101

We found that in another strain of *C. perfringens*, SM101, the *sipW* operon naturally contained insertion sequences, and SM101 produced adherent biofilms at 25 °C but not pellicle biofilms (Supplementary Fig. 6a, b). SM101 produced drastically lower amounts of extracellular BsaA proteins than HN13, suggesting that the insertion sequences diminish the expression of the *sipW* operon (Supplementary Fig. 6c). We introduced the plasmid containing *sipW-bsaA* genes from HN13 under the control of the xylose-inducible promoter into SM101. The HN13 *sipW-bsaA* expression enabled pellicle biofilm formation in SM101, indicating that high production of BsaA proteins was essential for pellicle biofilm formation (Supplementary Fig. 6d).

### BsaA polymer forms filamentous EPS

**Fig. 4:**
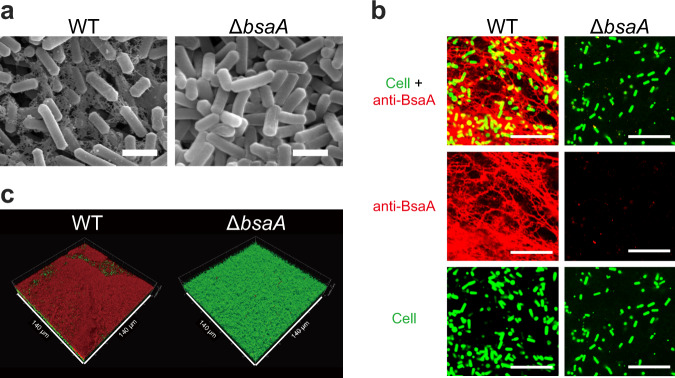
BsaA proteins form filamentous EPS and cover the surface of pellicle biofilms. **a** SEM images of WT or *bsaA* mutant cultures. Cells were grown at 25 °C for 2 days. Bar = 2 μm. **b** CLSM images of cells stained with SYTO9 DNA dye (green) and anti-BsaA antibodies labeled with HiLyte Fluor 555 (red). Cells were grown at 25 °C for 2 days. Bar = 20 μm. **c** 3D images of BsaA localization in the pellicle biofilm formed by WT cells and in the adherent biofilm formed by the *bsaA* deletion mutant. Cells were grown at 25 °C for 2 days. Fixed biofilms were probed with anti-BsaA antibodies (red). Biofilm cells were stained with Syto9 (green).

*C. perfringens* cells produce filamentous EPSs in pellicle biofilms (Fig. 4a)^7^. We have also reported that this filamentous EPS is resistant to DNase I treatment and slightly degraded by proteinase K treatment^7^. These findings suggest that the filamentous EPS structure consists of proteinaceous factors. We observed the biofilms of WT and the *bsaA* mutant by scanning electron microscopy (SEM) (Fig. 4a). WT cells were connected to each other by a filamentous EPS. Thin filamentous structures around cells were also observed by transmission electron microscopy (Supplementary Fig. 7). We did not observe filamentous EPS in the *bsaA* mutant cells, suggesting that BsaA proteins were required for filamentous EPS formation (Fig. 4a and Supplementary Fig. 7). To determine whether BsaA proteins were major components of the filamentous EPS, we visualized extracellular BsaA proteins by immunofluorescent staining. We observed a filamentous distribution of the anti-BsaA antibodies in the pellicle biofilms (Fig. 4b). The filamentous structure of BsaA proteins was localized around cells and connected cells to other cells (Fig. 4b). The deletion of *bsaA* abolished the filamentous structure, although a few nonspecific spot signals were observed (Fig. 4b). The BsaA-expressing plasmid restored the formation of the filamentous structure (Supplementary Fig. 8). These results suggested that extracellular BsaA proteins contributed to the cell-to-cell connection of pellicle biofilms. We also visualized the localization of BsaA proteins in pellicle biofilms. Figure 4c shows that BsaA proteins entirely covered the surface of WT pellicle biofilms at 25 °C. The *bsaA* mutant did not form pellicle biofilms at 25 °C but was able to form biofilms that adhered to the bottom surface (adherent biofilm) at both 25 and 37 °C. This finding indicated that BsaA polymers covered the top layer of the pellicle biofilm.

### BsaA protein facilitates antibiotic and oxidative stress resistance

**Fig. 5:**
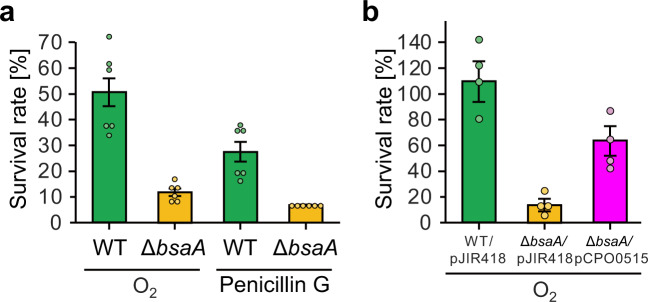
*bsaA* gene influences the response of *C. perfringens* biofilm to oxygen and penicillin G. **a** WT and *bsaA* mutant cells were grown for 3 days at 25 °C. Survival rates were calculated after exposure to oxygen or treatment with 50 µg ml^−1^ penicillin G for 20 h. **b** WT harboring pJIR418, *bsaA* mutant harboring pJIR418, and a *bsaA* mutant harboring pCPO0515 (*bsaA* complemented strain) were grown for 3 days. Survival rates were calculated after exposure to oxygen for 20 h. The bar graphs represent the mean values with standard errors.

Biofilm EPS facilitates not only biofilm architecture but also various types of stress resistance^1^. Thus, we hypothesized that filamentous EPS consisting of BsaA proteins could confer increased stress resistance of the cells in biofilms. We exposed the biofilm of WT or *bsaA* mutants to oxygen or the antibiotic penicillin G. Deletion of the *bsaA* gene significantly decreased the survival rate after treatment with oxygen and penicillin G (Fig. 5a). Complementation of the *bsaA* gene restored oxygen tolerance to the *bsaA* mutant (Fig. 5b). This result indicated that BsaA proteins promoted the tolerance of *C. perfringens* to antibiotics and oxidative stresses.

### Phenotypic heterogeneity in the biofilm

**Fig. 6:**
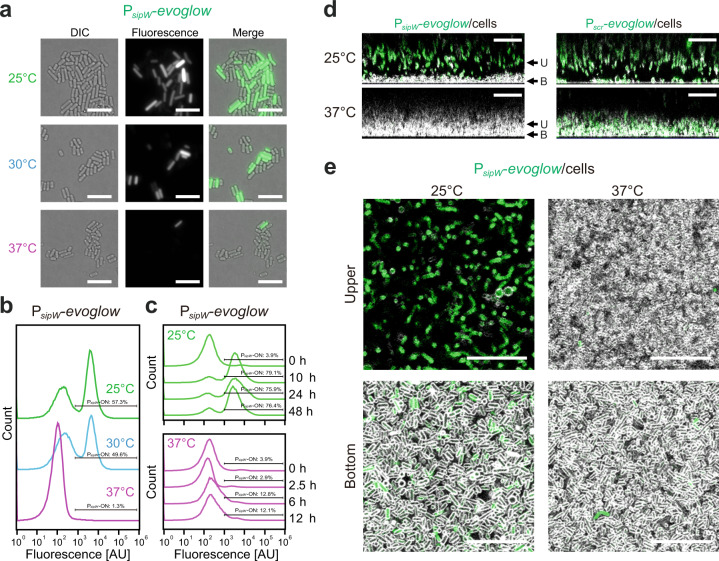
P_*sipW*_ expression is localized in pellicle biofilms and regulated by temperature. **a**, **b** P_*sipW*_-*evoglow* expression is bimodal and activated at lower temperatures. Fluorescence microscopy images (**a**) and FACS analysis (**b**) of planktonic P_*sipW*_-*evoglow* reporter cells at different temperatures. Cells were cultured for 1 day at 25, 30, or 37 °C. For FACS analysis, we counted 100,000 events for each sample. Bar = 10 µm. **c** Time-course analysis of *sipW* promoter activities in planktonic cell culture. We analyzed 100,000 events for each sample in the FACS analysis. The 10, 24, and 48 h time points at 25 °C and the 2.5, 6, and 12 h time points at 37 °C correspond to the exponential phase, early stationary phase, and late stationary phase, respectively. **d** Side views of the biofilm formed by the P_*sipW*_-*evoglow* reporter strain at different temperatures. The reporter strain was cultured for 1 day at 25 or 37 °C. We observed the biofilms by confocal laser microscopy. Cells were stained with the membrane staining dye FM4-64 (2.5 µg ml^−1^) (gray). The control promoter is the constitutively active gene *scr*, which exhibits a monomodal expression pattern. Bar = 20 µm. **e** Orthometric images of CLSM images corresponding to **d**. The *xy-*slice images of each *z* position indicated in **d** are shown. Bar = 20 µm.

We found that *sipW-bsaA* operon expression was stimulated at a lower ambient temperature (Fig. 2). To determine the single-cell level gene expression of the *sipW* operon, we constructed a fluorescent reporter under the control of the *sipW* promoter. Since *C. perfringens* is an anaerobic bacterium, we used the anaerobic fluorescent protein Evoglow for the reporter strains. Fluorescent microscopic observation and flow cytometry revealed that in the whole planktonic population, there were two types of cells (Fig. 6a, b): cells in which P_*sipW*_ is expressed (ON) and cells in which P_*sipW*_ is not expressed (OFF). As the temperature gradually increased, the number of P_*sipW*_-ON cells decreased. We still detected a small population of P_*sipW*_-ON cells at 37 °C, which is consistent with the northern blot results, where we detected *sipW* transcripts even at 37 °C (Fig. 1b). These results indicate that P_*sipW*_ expression is bimodal and that the subpopulation size of P_*sipW*_-ON cells is regulated by temperature. The ratio of P_*sipW*_-ON to P_*sipW*_-OFF cells did not change markedly at different growth time points (Fig. 6c).

Biofilm cells often show localized gene expression, which leads to phenotypic heterogeneity and multicellular behaviors. Thus, we were interested in the spatial distribution of bimodal P_*sipW*_ expression in biofilms. Cells were grown at 37 or 25 °C, and we visualized the spatial distribution of the gene expression in biofilms by confocal laser scanning microscopy (CLSM). At 37 °C, the whole population of cells was densely packed in the biofilm and attached to the glass surface. These cells rarely expressed P_*sipW*_, which was consistent with the flow cytometry results (Fig. 6a, b). In contrast, at 25 °C, P_*sipW*_-OFF cells were located next to the surface (Fig. 6d, e). In the upper fraction of the biofilms, the cell density was lower, which was a typical characteristic of cells in the pellicle biofilm, and P_*sipW*_-ON cells covered the P_*sipW*_-OFF cells (Fig. 6d, e). A reporter strain of the constitutive promoter P_*scr*_ revealed the fluorescent protein expression in the whole population (Fig. 6d). These results suggested an inverse regulation of adherence to the surface with P_*sipW*_ expression.

**Fig. 7:**
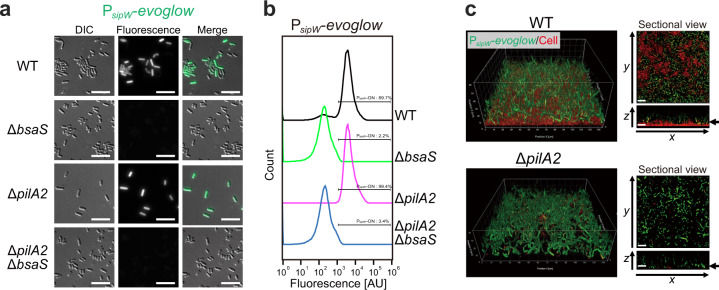
*pilA2* and *bsaS* are necessary for heterogeneous *sipW* expression. P_*sipW*_-*evoglow* expressing cells (green) are not located at the bottom. **a**, **b** Single-cell analysis of P_*sipW*_-*evoglow* expression in planktonic cells of Δ*pilA2* or Δ*bsaS*. **a** Fluorescent microscopy images. Bar = 10 µm. **b** Quantitative analysis of the *sipW* promoter by FACS analysis. We analyzed 100,000 events for each sample. **c** 3D and orthometric images of the localization of *sipW-*expressing cells in pellicle biofilms of WT or Δ*pilA2*. Cells were grown for 1 day at 25 °C and stained with the membrane stain FM4-64 (2.5 µg ml^−1^) (red).

The BsaR/BsaS two-component system is necessary for pellicle biofilm formation, the transcription of *sipW* mRNA, and BsaA protein production (Figs. 1a, b and 2a and Supplementary Fig. 2a). We also showed that the purified response regulator BsaR proteins specifically bound to the *sipW* promoter sequence (Supplementary Fig. 2b–d). Thus, the BsaR/BsaS two-component system positively controlled *sipW* promoter activity. To test the possibility that BsaS/BsaR was necessary for bimodal P_*sipW*_ expression, we analyzed the expression of the P_*sipW*_ fluorescent reporter in the *bsaS* sensor kinase mutant. We detected no P_*sipW*_ ON cells in the *bsaS* mutant strains during fluorescence microscope observations of more than 1000 cells (Fig. 7a, b). We observed that the *bsaS* mutant produced adherent biofilms at 25 °C and detected no P_*sipW*_ ON cells in the biofilm (Supplementary Fig. 9). These results suggested that the two-component system mediated P_*sipW*_ bimodal expression.

#### Type IV pili inhibit *bsaA* expression

In *C. perfringens*, type IV pili are known to be required for attachment to host cells^10^. Previously, we have shown that the *pilA2* gene, which encodes a major type IV pilin, is necessary for adherent biofilm formation^7^. To determine whether type IV pili could influence P_*sipW*_ expression and its spatial distribution within the biofilms, we used the *pilA2* mutant strain for fluorescent reporter analysis. Surprisingly, P_*sipW*_ expression was activated in the whole population of *pilA2* mutant cells, which did not adhere to the surface (Fig. 7c). We confirmed these results by fluorescence microscopy and flow cytometry (Fig. 7a, b). In addition, PilA2 overexpression inhibited pellicle biofilm formation (Supplementary Fig. 10). WT cells displayed bimodal P_*sipW*_ expression, and all cells of the *pilA2* mutant were in the ON state. We showed that BsaR/BsaS was also important for bimodal P_*sipW*_ expression. We found that in the *pilA2 bsaS* double mutant, all the cells were in the OFF state (Fig. 7a, b). These data showed that both PilA2 and the BsaR/BsaS two-component system were necessary for bimodal expression of P_*sipW*_.

#### RNase Y-dependent *pilA2* mRNA cleavage moderates matrix production

**Fig. 8:**
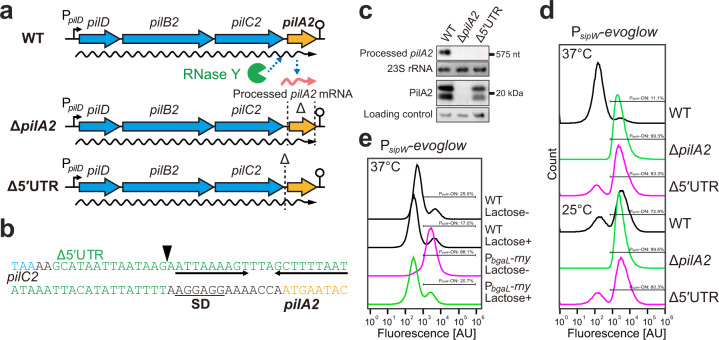
RNase Y-dependent *pilA2* mRNA cleavage is necessary for the temperature-dependent regulation of *sipW* expression. **a** Schematics of the posttranscriptional regulation model of the *pilA2* gene. We constructed a deletion mutant of the 5′ UTR of *pilA2*, which contains the cleavage site. **b** Nucleotide sequence of the upstream region of the *pilA2* gene. A triangle indicates a posttranscriptional cleavage site. Inverted arrows represent an inverted repeat sequence. The predicted ΔG value of the stem-loop structure is −4.60 kcal/mol. **c** Northern and western blotting related to *pilA2*. Cells were cultured to mid-exponential phase (OD_600_ = 1.0) at 37 °C. The 23S rRNAs stained with methylene blue are shown at the bottom as an RNA loading control. As a protein loading control, the protein that nonspecifically reacted with the antibodies are shown at the bottom. **d** Quantitative analysis of the *sipW* promoter in the *pilA2* 5′ UTR deletion mutant by FACS analysis. We analyzed 100,000 events for each sample. **e** Quantitative analysis of the *sipW* promoter in the RNase Y-depleted strain by FACS analysis. We analyzed 100,000 events for each sample.

The amount of PilA2 controls heterogeneous *bsaA* expression. Therefore, temperature-mediated *pilA2* expression induces the phenotypic change in biofilm formation. Previously, we found that *pilA2* mRNA was more highly accumulated at 37 °C than at 25 °C^7^. Since *sipW* expression was upregulated at 25 °C compared with 37 °C, *sipW* expression was inversely correlated to *pilA2* expression. We showed that the accumulation of *pilA2* mRNA was posttranscriptionally regulated by mRNA processing, mediated by an endoribonuclease, RNase Y^33^. The *pilA2* gene was transcribed as *pilD-pilB2-pilC2-pilA2* polycistronic mRNA from the *pilD* promoter, and the *pilA2* 5′ UTR was then cleaved by RNase Y to generate the *pilA2* monocistronic mRNA. The half-life of the *pilA2* monocistronic mRNA is longer than *pilD-pilB2-pilC2-pilA2* polycistronic mRNA, which facilitates the upregulation of PilA2 protein expression^33^. We hypothesized that the posttranscriptional regulation of *pilA2* generates a subpopulation of *bsaA*-expressing cells. To test this notion, we constructed a deletion mutant of the *pilA2* 5′ UTR (Fig. 8a, b). Because we had already determined the 5′ end of the *pilA2* monocistronic mRNA, we decided to delete the *pilA2* 5′ UTR sequence containing the 5′ end but not the Shine-Dalgarno sequence. We confirmed that deletion of the *pilA2* 5′ UTR drastically decreased the *pilA2* monocistronic mRNA and slightly decreased the amount of PilA2 protein (Fig. 8c). These results indicated that the *pilA2* 5′ UTR was necessary for the generation of *pilA2* monocistronic mRNA and the marked accumulation of the PilA2 protein. To determine the effect of the differential expression of PilA2 in the *pilA2* 5′ UTR deletion mutant on *sipW* expression, we monitored the expression of the P_*sipW*_ reporter in this mutant grown at different temperatures. The *pilA2* 5′ UTR mutant showed a bimodal expression pattern of P_*sipW*_, and the ratio of the ON and OFF cells was not changed at different temperatures (Fig. 8d). This finding indicates that the 5′ UTR sequence of *pilA2* was required for the regulation of P_*sipW*_ expression in response to temperature. Moreover, we used a knockdown mutant strain of the *rny* gene encoding RNase Y, in which *rny* gene expression was under the control of the lactose-inducible promoter, P_*bgaL*_. As shown in Fig. 8e, in the *rny* knockdown mutant (P_*bgaL*_-*rny* without the lactose inducer), the P_*sipW*_-ON population was increased at 37 °C. These results suggested that the expression of *pilA2* regulated by RNase Y-dependent cleavage of the *pilA2* 5′ UTR affected the ratio of bimodal P_*sipW*_ expression in the population.

## Discussion

Recently, many clostridial bacteria have been reported to be capable of building biofilms^36^. The clostridial biofilm matrix, similar to other bacterial biofilms, is composed of proteins, DNA, and polysaccharides^4,7,37,38^. However, the specific biofilm EPS produced by clostridial bacteria has been unclear. In the present study, we identified that the BsaA protein is necessary for building pellicle biofilms. BsaA is an extracellular protein that forms a filamentous structure, which greatly facilitates the morphological change in *C. perfringens* biofilms in response to temperature.

Western blotting and fluorescent immunostaining of BsaA protein indicated that the BsaA protein formed HMW polymers in the extracellular fraction. The amino acid sequence of BsaA contained a putative signal peptide sequence. In contrast, in the *sipW* deletion strain, BsaA protein monomers accumulated in the intracellular fraction, which suggested that the SipW signal peptidase cleaved an N-terminal signal peptide sequence and was necessary for extracellular secretion of BsaA proteins. Moreover, extracellular secretion of BsaA proteins was likely to induce the polymerization of BsaA proteins. Bacterial signal peptidases are classified into three types: signal peptidase I, signal peptidase II, and prepilin peptidase^39^. The *C. perfringens* genome is predicted to contain eight genes encoding signal peptidases: six signal peptidase I, one signal peptidase II, and one prepilin peptidase. SipW is expected to be a signal peptidase I. In *B. subtilis*, SipS and SipT are major signal peptidases since the *sipS* and *sipT* double mutant is not viable^40^. The *C. perfringens* CPE0597 gene, a predicted orthologous gene of *B. subtilis sipS* and *sipT* (35.9% and 37.9% identity, respectively), may be responsible for the cleavage of the essential preproteins. Adding further support, the *B. subtilis* SipW, a homolog of *C. perfringens* SipW (33.5% identity), is not essential for general protein secretion and has two major substrates, TapA and TasA, which is necessary for biofilm formation^11,41^. Thus, we predicted that *C. perfringens* SipW could also function as a specific signal peptidase for biofilm formation by catalyzing the cleavage of BsaA proteins to secrete proteins into the extracellular medium.

Some proteins that form polymers require accessory proteins for their polymerization^41–43^. *bsaB* and *bsaC* genes, which are located in the *sipW* operon, encode putative extracellular proteins, respectively. We found that the polymerization of BsaA was not inhibited by the deletion of *bsaB* and *bsaC* genes, although the amount of BsaA protein decreased slightly (Fig. 2a). Furthermore, recombinant BsaA proteins expressed in *E. coli*, which have no homologous genes of *bsaB* and *bsaC*, formed stable HMW polymers that were resistant to high-SDS and formic acid treatments (Fig. 2b). Thus, we suggested that the polymerization of BsaA proteins did not require BsaB and BsaC proteins.

The polymerization of BsaA proteins did not require a putative sortase-encoding gene (*srtB*), which was located in the 5′ flanking region of the *sipW* gene (Supplementary Fig. 5). Sortases frequently recognize C-terminal sorting sequences, an LPXTG motif. We did not predict any similar sequences of LPXTG motifs in the BsaA amino acid sequence. Although BsaA polymers were detected at low concentrations in whole cell fractions, BsaA polymers were mainly detected in the extracellular fraction (Fig. 2a). These results suggest that BsaA proteins do not bind tightly to cell walls and that the polymerization of BsaA protein is unlikely to need sortase enzymes.

*B. subtilis* TasA is a major component of the biofilm matrix that forms amyloid fibers, which provide structural integrity^44^. *C. perfringens* BsaA has several similar characteristics to TasA: (1) both proteins have a number of beta-strands in their amino acid sequences; (2) extracellular secretion of both proteins depends on the SipW homolog; and (3) both proteins form polymers in the extracellular medium. To compare the functional similarity of BsaA and TasA, we cultured *C. perfringens* WT and Δ*bsaA* on media containing Congo Red, which is known to bind amyloid fibers^44^. The colonies of WT and *bsaA* complemented strains were not stained with Congo Red (Supplementary Fig. 11a). Moreover, we tested purified BsaA proteins for the assay using Congo Red and Thioflavin T, which bind to amyloid fibers and cause an increase in absorbance and fluorescence, respectively. The addition of Congo Red and Thioflavin T to the purified his-tagged BsaA proteins did not show any increased absorbance and fluorescence, respectively, compared with a protein, BsaR (Supplementary Fig. 11b, c). Surprisingly, the SDS-PAGE procedure (i.e., addition of SDS, boiling, and reduction), as well as the 10% SDS or 20% formic acid treatment, did not ablate the formation of BsaA polymers. Although there is a possibility that the BsaA polymer is an amyloid fiber as TasA, both proteins have different biochemical characteristics. Protein structure determination of BsaA is needed to elucidate how BsaA polymers possess such extreme resistance and should be addressed in future studies.

Furthermore, we found that P_*sipW*_ expression is bimodal in *C. perfringens* populations and the population size is increased by lower temperature. Our data suggest that the BsaR/BsaS two-component system is required for P_*sipW*_ expression. *bsaRS* is reported to be involved in the expression of citrate metabolism genes^45^. This result implies that extracellular citrate influences *sipW-bsaABCRSD* operon expression. However, the addition of citrate to the media did not induce the expression of P_*sipW*_ (Supplementary Fig. 12). The addition of 10 mM citrate slightly inhibited P_*sipW*_ expression. We also observed that a higher concentration citrate inhibited cell growth, likely explaining the slight suppression of P_*sipW*_ expression. These results suggest that citrate does not act as the main regulator of pellicle biofilm formation under our experimental conditions.

Whereas the signals recognized by *bsaS* are still unclear, we found that PilA2 expression inhibited *bsaA* expression in a BsaS-dependent manner. PilA2 is a type IV pilin in *C. perfringens* and is necessary for adherence to host cell surfaces^46^. Recently, the type IV pilin PilA has been shown to interact directly with a two-component system, which mediates cell-cycle initiation in *Caulobacter crescentus*^47^. The two-component system senses a 17-amino acid sequence, the transmembrane domain of PilA. Although *C. perfringens* PilA2 bears no sequence similarity to *C. crescentus* PilA, anchoring of the PilA2 monomeric proteins to the membrane during translocation may mediate temperature-responsive *sipW-bsaA* expression.

The amount of *pilA2* monocistronic mRNA was increased at 37 °C compared with 25 °C, which is consistent with the converse expression of the *sipW* operon. Therefore, the temperature-dependent regulation of PilA2 expression should be necessary for the regulation of P_*sipW*_ expression and biofilm morphology. We also found that cleavage of the *pilA2* 5′ UTR by RNase Y was critical and heterogeneous expression of *bsaA*. A stem-loop structure was predicted in the 5′-proximal RNA structure of the *pilA2* monocistronic mRNA (Fig. 8b), which might stabilize the transcript. The deletion of a sequence containing the predicted stem-loop structure eliminated *pilA2* monocistronic mRNA, whereas PilA2 proteins were still detected by western blotting. Thus, the *pilA2* 5′ UTR was necessary for the generation of *pilA2* monocistronic mRNA and temperature-dependent regulation of *pilA2*. However, *pilA2* mRNA was not necessarily required for the production of PilA2 proteins, which could also be translated from *pilD-pilB2-pilC2-pilA2* polycistronic mRNA.

In summary, we identified extracellular protein BsaA serving as a biofilm matrix, which increases tolerance to oxygen and antibiotics of *C. perfringens*. The bimodal expression of *bsaA* could lead to the division of labor in *C. perfringens*. This heterogeneity is modulated by temperature, resulting in morphological changes in biofilm. Temperature is an environmental cue, and the temperature inside the host is generally higher than in the environment outside the host. The expression of *sipW-bsaA* was elevated at 25 °C, the temperature outside the host. Under this condition, *C. perfringens* cells are more likely to be exposed to external stresses such as oxygen. Furthermore, the heterogeneous expression of P_*sipW*_ facilitates the maintenance of a small population of BsaA-producing cells at 37 °C, which could possess higher tolerance than BsaA-nonproducing cells. If cells are rapidly excluded from inside the host, they must quickly adapt to an oxygen-rich environment for survival. Therefore, we suggest that the heterogeneous expression of the biofilm matrix in *C. perfringens* acts as a bet-hedging strategy.

## Methods

### Bacterial strains and growth conditions

The bacterial strains and plasmids used in this study are listed in Supplementary Table 2. *C. perfringens* strains were routinely cultured in Gifu anaerobic medium (GAM) (Nissui Co. Japan) agar plates under anaerobic conditions using an Anaeropack system (Mitsubishi Gas Chemical Co. Inc., Tokyo, Japan) or PGY or GAM liquid broth. *E. coli* JM109 and M15 were cultured in LB medium. When necessary, antibiotics were supplemented in the media; for *C. perfringens*, 20 μg ml^−1^ chloramphenicol was used, and for *E. coli*, 100 μg ml^−1^ ampicillin, 40 μg ml^−1^ chloramphenicol, and 20 μg ml^−1^ kanamycin were used.

### Biofilm assay

Overnight culture of *C. perfringens* strains in PGY medium (3% proteose peptone no. 3, 2% glucose, 1% yeast extract, and 0.1% sodium thioglycolate) was diluted 1:100 with 2 or 4 ml of GAM broth in 24- or 6-well polystyrene plates. These cells were grown anaerobically for 1–3 days. To assess the resistance of biofilms to antibiotics or oxygen, we cultured the cells in 1 ml GAM broth in 24-well polystyrene plates. After incubation at 25 °C for 3 days, we removed the supernatant and washed the biofilms 2× with phosphate-buffered saline (PBS). The biofilms were then placed in 200 µl of aerobic PBS or GAM containing 50 μg ml^−1^ penicillin G, and were aerobically incubated at 37 °C for 20 h. After 20 h, we added 800 µl of PBS and suspended the biofilms by thorough pipetting. The variable cells in each 100 µl of the suspension were measured using BacTiter-Glo™ reagent (Promega, Madison, WI, USA) according to the manufacturer’s instructions.

### Oligonucleotides

The oligonucleotides used in this study are listed in Supplementary Table 3.

### Strain and plasmid construction

We constructed the gene deletion mutants using a galactose counter selection system^48^. Approximately 1 kb of the 5′ and 3′ flanking regions of the target gene were amplified and joined by overlap PCR. The purified DNA fragments were digested with SalI and BamHI. These fragments were ligated and cloned into the SalI/BamHI site of pCM-GALK, and the resulting plasmid was introduced into *C. perfringens* HN13 via electroporation. Agar plates with 20 μg ml^−1^ chloramphenicol or 3% galactose were used to isolate the strains. The obtained strain was confirmed by PCR and DNA sequencing.

We constructed lactose-inducible SipW-FLAG or BsaA expression plasmids as follows. To construct the FLAG-fusion protein expression vector, we amplified *lipA* transcriptional terminator sequences using the primer set containing codon sequences of the FLAG (DYKDDDK) fragment. This DNA fragment was digested and ligated into the SalI/HindIII site of the *E. coli-C. perfringens* shuttle vector pJIR418. Subsequently, DNA fragments containing *sipW* or *bsaA* genes were amplified and digested with BglII/SalI or BamHI/SalI, respectively. The sequences containing the *bgaR* ORF and the *bgaL* promoter, a lactose-inducible promoter^49^, were amplified and digested with SacI/BamHI. These fragments were ligated and cloned into the pJIR418 vector containing the *lipA* terminator.

Xylose-inducible SipW and BsaA expression plasmids were constructed as follows. DNA sequences of *sipW* or *sipW-bsaA* were amplified and digested with SalI/BglI. The purified DNA fragments were cloned into pXCH^50^.

We constructed recombinant His-tagged protein expression vectors as follows. A *bsaA* ORF that did not contain the signal peptide sequence was amplified and digested with NcoI/BamHI. The purified DNA fragments were cloned into pQE60. BsaR-6×His-expressing plasmids (pQE60-bsaR) were similarly constructed.

To construct the fluorescent reporter plasmid, we used the anaerobic fluorescent protein Evoglow-C-Pp1, the codon usage of which has been optimized for efficient expression in *Clostridia*. We determined the transcriptional start site of the *sipW* gene by 5′ RLM-RACE (Supplementary Fig. 1). First, we cloned a strong intrinsic transcriptional terminator located downstream of the *lipA* gene into the SalI/HindIII site of pJIR418. To enhance the stability of the mRNA and the translational efficiency, we used the 5′ UTR and leader sequence of *colA*. Previously, we showed that the processed form of *colA* mRNA is highly expressed in *C. perfringens*^18^. Fragments containing the promoters of *scr* or *sipW*, the *colA* 5′ UTR and leader sequence, and *evoglow-C-Pp1* were amplified and cloned into pJIR418 with a *lipA* terminator. The resultant plasmids, pCPE2002 and pCPE2005, showed fluorescence sufficiently bright for analysis using flow cytometry and CLSM. The promoterless plasmid pCPE2001 was used as a negative control.

We constructed a xylose-inducible *pilA2* plasmid as follows. The DNA fragments containing *pilA2* CDS and pXCH were amplified by PCR. These fragments were used for InFusion cloning (Takara Bio, Japan).

All resulting plasmids were confirmed by PCR, restriction enzyme cleavage, or DNA sequencing.

### Random transposon mutagenesis and screening

To construct a transposon library of *C. perfringens* str 13, we used the EZ-Tn5 transposome system (Lucigen, Middleton, WI). First, we cloned the *ermBP* gene into the EcoRI-HindIII site of pMOD-2 plasmid. To generate the transposome, we incubated the transposase with the DNA fragment of PvuII-digested pMOD-2-ermBP according to the manufacturer’s instruction. The resultant transposome was introduced into *C. perfringens* by electroporation, and the transposon mutants were selected on GAM plates with 50 µg µl^−1^ erythromycin. To screen for mutants deficient in pellicle biofilm formation, we cultured the mutants in GAM medium in 96-well plates and visually determined which mutants were able to form pellicle biofilms. The pellicle biofilm-negative mutants were further confirmed by culturing in GAM medium in 24-well plates. Of 51 transposon mutants that did not form a pellicle biofilm, we randomly chose 42 mutants for sequencing. The sequences flanking the *ermBP*-containing transposon were determined by Sanger sequencing with 1 µg of each genomic DNA and 5 pmol of primers NOB-0301 or NOB-0302. We succeeded in sequencing 34 genomic DNAs among these mutants.

### Northern blot analysis

The *C. perfringens* strains were cultured overnight in PGY medium. The cells were inoculated in fresh GAM broth to reach an OD_600_ of 0.1. The cultures were incubated for 2 h at 37 °C or for 8 h at 25 °C to reach the mid-exponential phase of growth (OD_600_ ≈ 1.0). Total RNA was extracted from *C. perfringens* using the SV Total RNA Isolation System according to the manufacturer’s instructions (Promega, Tokyo, Japan). Northern blot analysis was performed as previously described. Digoxigenin-labeled DNA probes were generated using DIG-High Prime (Roche). The primers used for amplification of the template DNAs are listed in Supplementary Table 3. All the blots presented as part of the same series were derived from the same experiment and were processed in parallel. Original blots are shown in Supplementary Information.

### 5′ RLM-RACE

The 5′ ends of the transcripts were determined by 5′RLM-RACE. Total RNA (3 μg) was treated with or without 20 units of TAP in 10 μl of reaction buffer at 37 °C for 1 h, and then 2 μl of TAP-treated or untreated RNA (relative to 0.6 μg RNA) was incubated with the 5′ RACE adapter and with 5 units of T4 RNA ligase (Life technologies) at 37 °C for 1 h. The total RNA ligated to the adapter (relative to 0.12 μg RNA) was used as a template for reverse transcription. Reverse transcription was performed with PrimeScript reverse transcriptase (Takara Bio) and 25 μM random hexamers. We amplified cDNAs by nested PCR using the primers listed in Supplementary Table 3. The amplified DNA products were cloned into *E. coli* using the pUC18 vector. The resulting vector sequences were determined by DNA sequencing using M13 primers.

### Western blot analysis

We isolated extracellular and whole cell proteins from 1-day-old biofilms in accordance with a previous protocol^33^. The culture supernatants were collected via centrifugation at 5000 × *g* for 5 min and incubated with 20% trichloroacetic acid. After washing with ice-cold acetone, the precipitates were dissolved in SDS sample buffer (50 mM Tris-HCl, pH 6.8, 10% glycerol, 2% SDS, 2.5% mercaptoethanol, and 0.1% bromophenol blue). The cells were incubated with 1 μg ml^−1^ lysozyme in PBS for 5 min and then dissolved in SDS sample buffer. After boiling for 10 min, the protein samples (OD_600_ = 0.002) were separated by SDS-PAGE and electroblotted onto PVDF membranes. The membranes were blocked with 2.5% skim milk in Tris-buffered saline containing 0.05% Tween 20. BsaA and FLAG proteins were probed with anti-BsaA and anti-DYKDDDK antibodies (Wako) diluted 1:5000. These antibodies were labeled with anti-rabbit IgG antibodies (GE Healthcare) diluted 1:20000. The bound antibodies were labeled with Immunostar LD (Wako, Osaka, Japan) and detected using a FUSION-SL7-400 Chemiluminescence Imaging System (Vilber-Lourmat, Marne-la-Vallée, France). All the blots presented as part of the same series were derived from the same experiment and were processed in parallel. Original blots are shown in Supplementary Information.

### Purification of His-tagged protein

We purified His-tagged proteins from *E. coli*. *E. coli* M15 harboring pREP4 (Qiagen) and pQE60-bsaA or pQE60-bsaR was grown in 100 ml of LB broth containing 100 μg/ml ampicillin and 25 μg/ml kanamycin to the mid-exponential phase. After adding 1 mM isopropyl-β-D-thiogalactopyranoside, the cells were incubated for an additional 5 h and then harvested by centrifugation. The cells were then resuspended in 5 ml of lysis buffer containing 50 mM NaH_2_PO_4_, pH 8.0, 300 mM NaCl, and 10 mM imidazole and sonicated using a sonicator Bioruptor (SONIC BIO Corporation, Kanagawa, Japan). After adding 300 μl of His-Select Nickel Affinity Gel (Sigma), the cell extract was gently mixed at 4 °C for 1 h. The gels that bound to the His-tagged protein were washed twice with lysis buffer containing 20 mM imidazole. The His-tagged protein was then eluted with lysis buffer containing 250 mM imidazole and dialyzed against dialysis buffer containing 20 mM Tris-HCl, pH 7.5, 100 mM NaCl, 1 mM EDTA, and 50% glycerol at 4 °C for 16 h.

### SEM

Cells were anaerobically grown in 4 ml of GAM broth in six-well plates containing 12-mm coverslips (Fisher). After incubation for 2 days at 25 °C, the coverslips were transferred to a fresh 24-well plate, and the cells attached to the coverslips were fixed in 2.5% glutaraldehyde-10 mM sodium phosphate (pH 7.5) overnight. The samples were washed twice with 10 mM sodium phosphate (pH 7.5) and dehydrated in 50, 70, 90, and 99.5% ethanol. Subsequently, the ethanol was replaced with 50% ethanol-50% isoamyl acetate and 100% isoamyl acetate. The coverslips were mounted onto aluminum stubs, dried using a critical-point dryer (HCP-2; Hitachi Ltd., Japan), and subsequently sputter-coated with platinum using an E-1030 ion sputtering machine (Hitachi Ltd., Japan). The EPS filaments in biofilms were observed using SEM (HITACH-S-4200) (Hitachi Ltd., Japan).

### Immunostaining and CLSM

WT and Δ*bsaA C. perfringens* were inoculated into 200 μl of GAM broth in multiwell glass-based dishes and anaerobically cultured for 1 day at 25 °C. Cells were fixed with 4% formaldehyde for 30 min and washed with PBS. After blocking with 1% BSA for 30 min, BsaA proteins were probed with anti-BsaA antibody diluted 1:100 for 1 h. The bound antibody and cells were labeled with 10 μg ml^−1^ of HiLyte Fluor 555-conjugated anti-rabbit IgG (AnaSpec, Fremont, CA, USA) and 1.67 μM Syto9 (Molecular Probes, Invitrogen, Eugene OR, USA). After washing with PBS, to visualize the samples, we used an LSM780 laser scanning microscope equipped with a 63×/1.4 numerical aperture Plan-Apochromat objective (Carl Zeiss, Jena, Germany). The biofilms were irradiated with 488- and 543-nm lasers, and 500–530-nm and 555–600-nm emission lights were used to build the images. We used ZEN (Carl Zeiss, Jena, Germany) and Imaris software (Bitplane, Switzerland) to process the images.

### Flow cytometry

Biofilms were disrupted by thorough pipetting, and the cells were collected by centrifugation. The cells were suspended in 4% formaldehyde/PBS for 10 min. After fixation, the cells were washed with PBS, resuspended in PBS, and stored at 4 °C. These cells were measured on an SH800 flow cytometer (Sony Corp., Tokyo, Japan). The expression of P*sipW* was analyzed using all events detected by flow cytometry. We confirmed that no signals were detected in the suspension (PBS). The fluorescence was excited with a 488-nm laser, and emission was subsequently detected with a 525/50-nm filter. For each sample, data were recorded for 100,000 counts. Data analysis was performed with SH800 software and FlowJo V10.7.

### Reporting summary

Further information on research design is available in the Nature Research Reporting Summary linked to this article.

## Supplementary information

Supplementary Information

Reporting Summary

## Data Availability

The datasets that support the findings of the current study are available from the corresponding author on reasonable request.
