# Supplementary material for: Temperature-regulated heterogeneous extracellular matrix gene expression defines biofilm morphology in Clostridium perfringens
Source: NPJ Biofilms Microbiomes. 2020 Jul 31;6:29. doi: 10.1038/s41522-020-00139-7 (PMC7395162; doi:10.1038/s41522-020-00139-7)
Supplement: Supplementary file 1 — Supplementary Information [file 41522_2020_139_MOESM1_ESM.pdf]

Supplementary information

Temperature-regulated heterogeneous extracellular matrix gene expression defines biofilm morphology in *Clostridium perfringens*

Nozomu Obana, Kouji Nakamura, Nobuhiko Nomura

Supplementary Figure 1-12

Supplementary Table 1-3

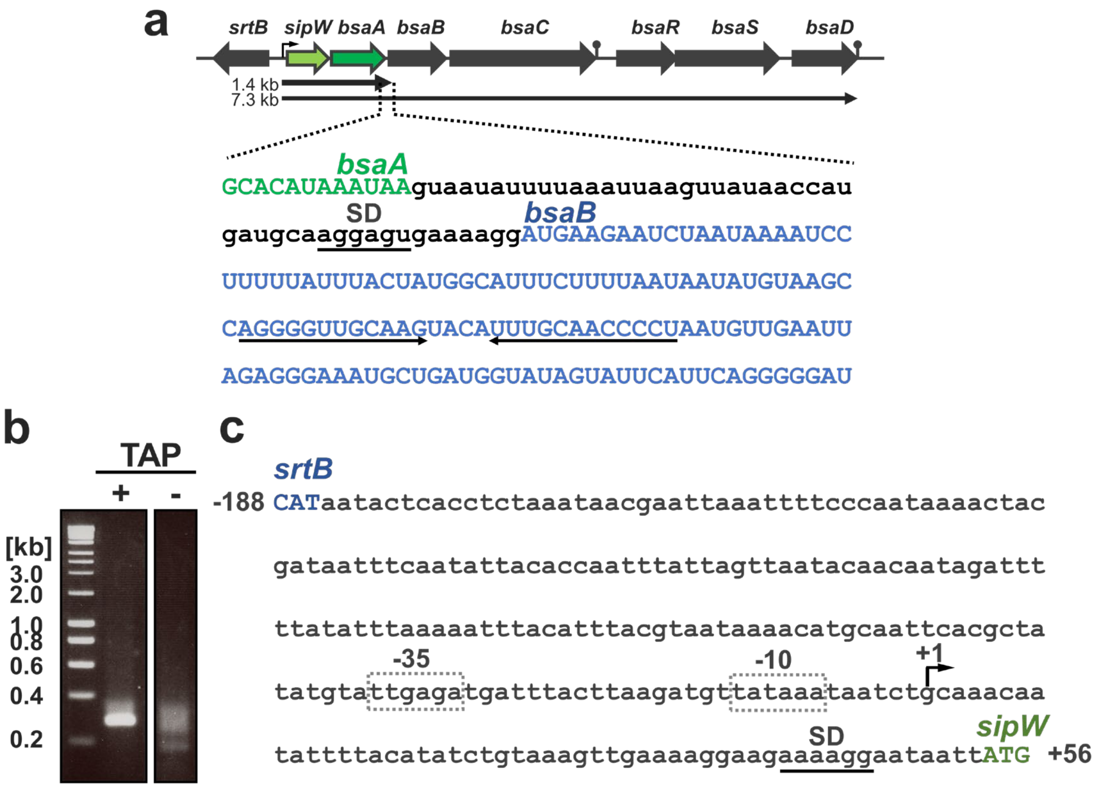

Supplementary Figure 1. Identification of a transcriptional start site of *sipW* operon. (a) Schematics of the *sipW* operon and nucleotide sequence downstream of the *bsaA* gene. Inverted arrows show inverted repeat sequences. (b) Determination of the 5′ end of *sipW* mRNA. Total RNA was left untreated or treated with TAP to distinguish primary transcripts from processed transcripts. PCR products amplified using primers for detection were resolved on a 2% agarose gel and stained with ethidium bromide. (c) Nucleotide sequence of the *sipW* promoter region. The “+1” label and bent arrow indicate the 5′-terminal site of the *sipW* transcripts detected by 5′ RLM-RACE. Putative -10 and -35 sequence regions are boxed in dashed lines. The “+1” sign indicates the 5′-terminal sites of the *sipW* transcripts detected by 5′ RLM-RACE.

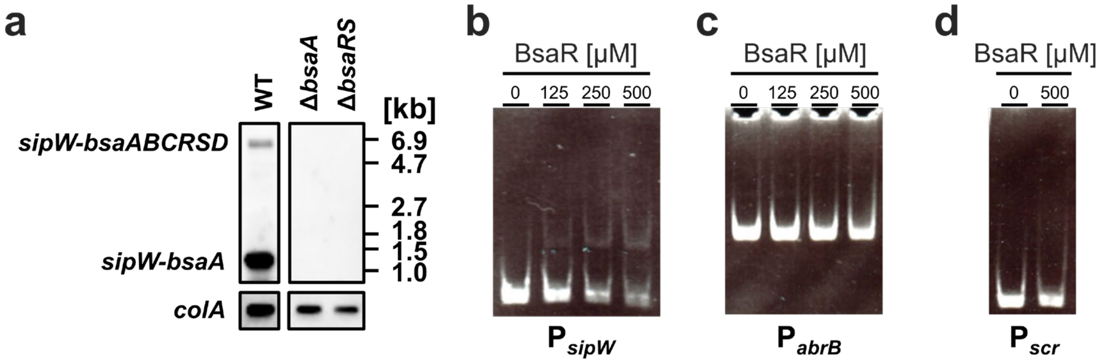

Supplementary Figure 2. BsaRS two-component system regulates the *sipW* expression. (a) Northern blotting of *sipW-bsaA*. Cells were grown at 37°C for 2 h. Total RNA (1 μg) was separated on 1.2% denaturing agarose and probed with *sipW-bsaA*-specific DIG-labeled probes. As loading controls, the northern blotting of *colA*, the kappa-toxin (collagenase) gene, is shown. (b-d) Gel mobility shift assay with purified BsaR-His6 proteins. DNA fragments (50 ng) corresponding to the *sipW* promoter (b), *abrB* promoter (c) and *scr* promoter (d) were incubated with 0, 125, 250 or 500 μM protein. Protein-DNA complexes were detected in the mixture of the BsaR proteins and the *sipW* promoter sequence.

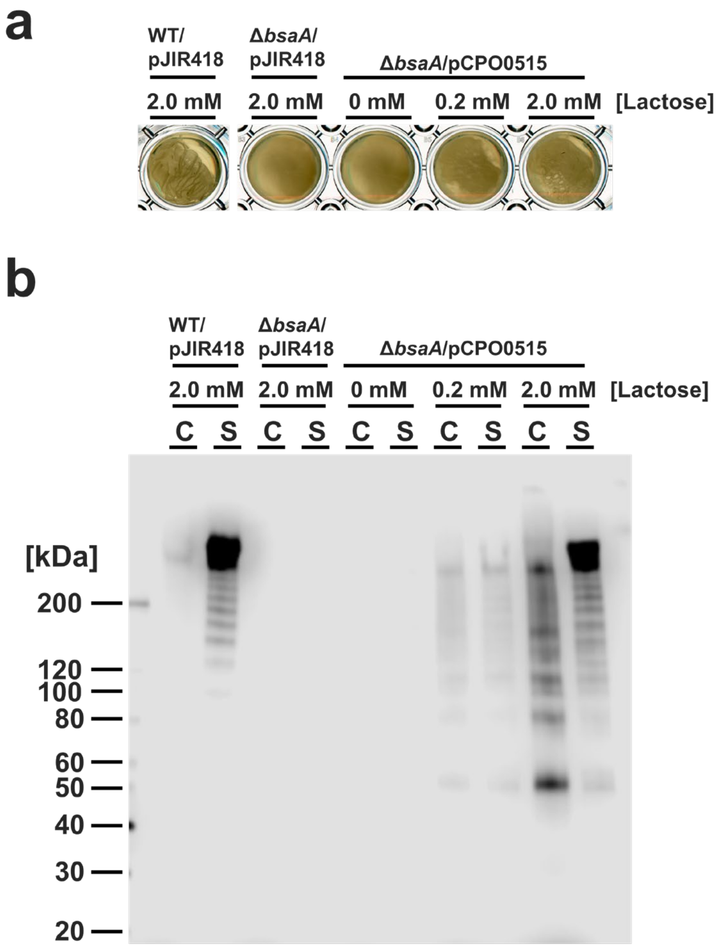

Supplementary Figure 3. Complementation of *bsaA* restores pellicle biofilm formation. (a) Pellicle biofilm formation of strains harboring plasmids expressing the *bsaA* gene. Cells were grown at 25°C for 2 days with or without lactose. The photographs show the pellicle biofilm after picking by gentle pipette aspiration. The transcription of the *bsaA* gene in the complementation plasmid pCPO0515 is under the control of the lactose-inducible promoter *bgaL*. (b) Western blotting of BsaA proteins. Whole cell proteins (C) and supernatant proteins (S) were extracted from the cultures corresponding to Supplementary Figure 3a. The proteins were separated with 4-15% SDS-PAGE and probed with anti-BsaA antibodies. Each lane contained protein at O.D. 600 unit=0.002.

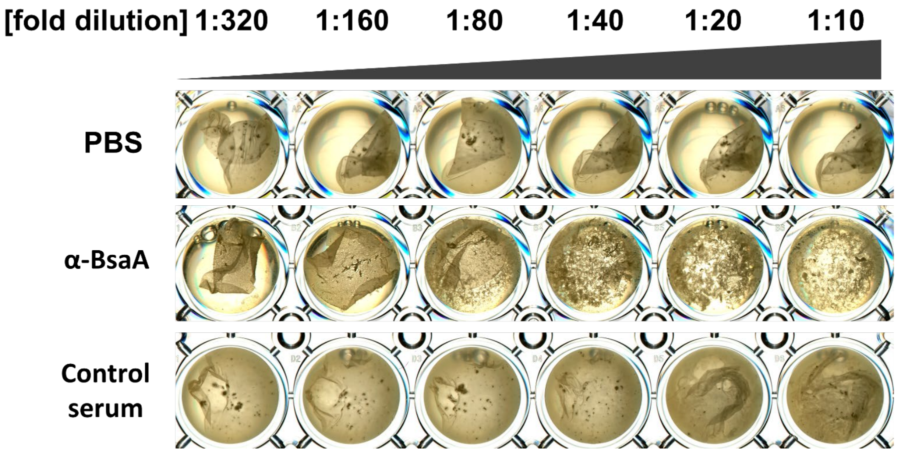

Supplementary Figure 4. Anti-BsaA antibody inhibits pellicle biofilm formation. An anti-BsaA antibody was added to the culture medium at a ratio indicated prior to incubation. PBS and rabbit serum of nonspecific to BsaA were used as negative controls. *C. perfringens* wild type (WT) cells were anaerobically grown at 25°C for 2 days. The photographs show the pellicle biofilm after picking by gentle pipette aspiration.

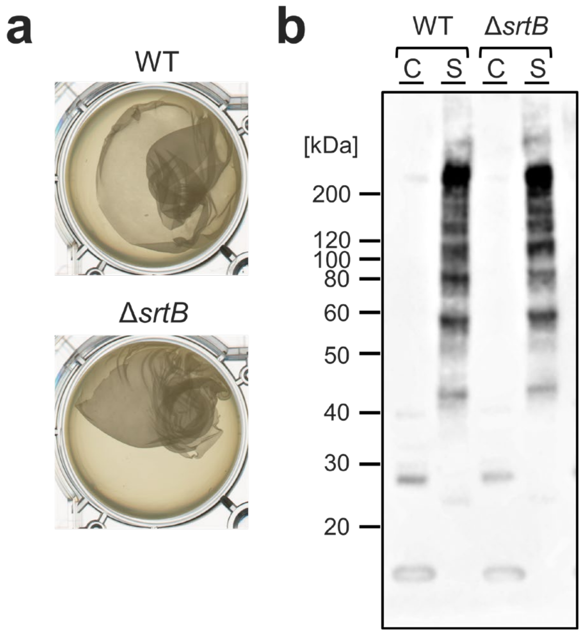

Supplementary Figure 5. *srtB* is not necessary for pellicle biofilm formation and BsaA protein expression. (a) Pellicle biofilm formation of WT and Δ*srtB*. The photographs show the pellicle biofilm after picking by gentle pipette aspiration. (b) Western blotting of BsaA proteins. Whole cell proteins (C) and supernatant proteins (S) were extracted from the cultures of WT and Δ*srtB*. The proteins were separated with 4-15% SDS-PAGE and probed with anti-BsaA antibodies. Each lane contains proteins at O.D. 600 unit=0.002.

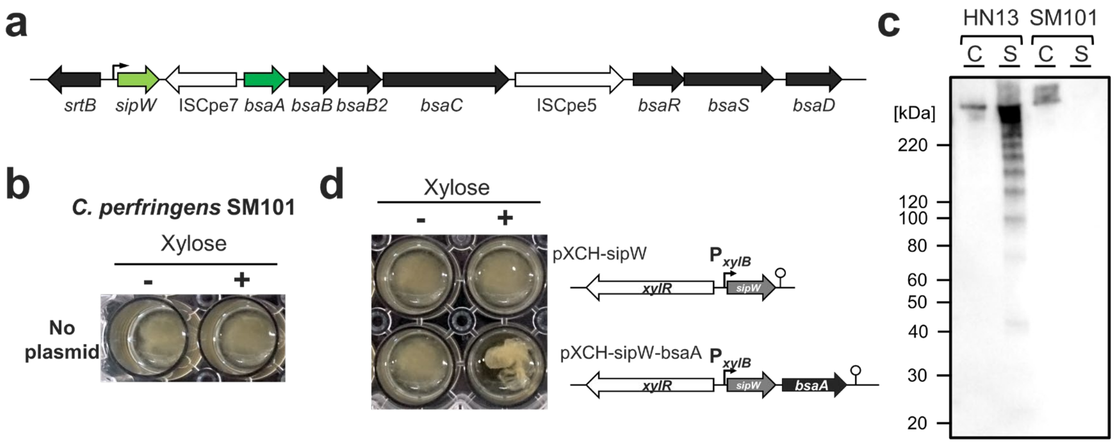

Supplementary Figure 6. Overexpression of *sipW-bsaA* from HN13 allows pellicle biofilm formation in SM101. (a) A schematic image of the *sipW* operon in *C. perfringens* type F strain SM101. An insertion sequence was naturally integrated into the *sipW* operon of the SM101 genome. (b) Pellicle biofilm formation of SM101. SM101 was deficient in pellicle biofilm formation. Cells were anaerobically grown at 25°C for 2 days. (c) Western blotting of BsaA proteins in HN13 and SM101. Cell extracts (C) and culture supernatants (S) were isolated from HN13 and SM101 cells grown at 25°C for 8 h to reach the mid-exponential phase. Protein samples (OD600 unit=0.004/lane) were separated on a 4-12% gradient SDS-polyacrylamide gel. BsaA proteins were detected with anti-BsaA antisera. (d) Pellicle biofilm formation of SM101 harboring the *sipW-bsaA* expression plasmid. Schematics show the plasmid construction for *sipW* or *sipW-bsaA* overproduction. The *sipW* or *sipW-bsaA* derived from *C. perfringens* type A strain HN13 in the plasmids were under the control of the xylose-inducible promoter *xylB*. Xylose (0.01%) was added to the medium prior to incubation for the induction of the gene expression. The *sipW-bsaA* expression restored pellicle biofilm formation in SM101. Cells were anaerobically grown at 25°C for 2 days. The photographs show the pellicle biofilm after picking by gentle pipette aspiration.

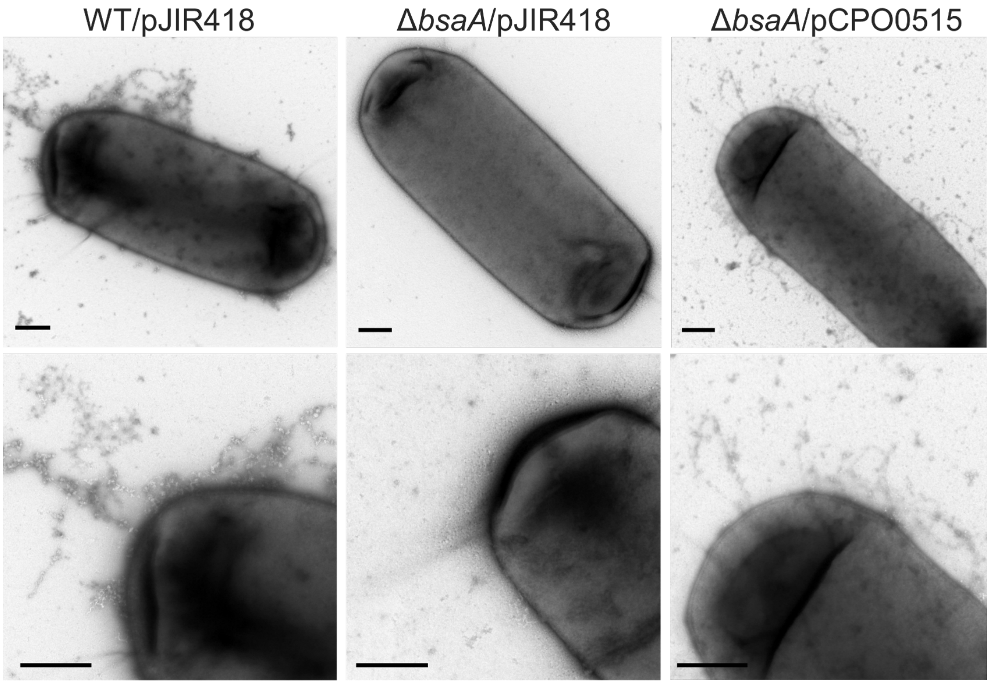

Supplementary Figure 7. Transmission electron microscopic images of filamentous EPS. Cells were anaerobically grown in GAM broth containing 2 mM lactose at 25°C for 2 days. Filamentous structures were observed in the periphery of WT cells or Δ*bsaA* cells complemented with the *bsaA*-expressing plasmid. Bar=400 nm.

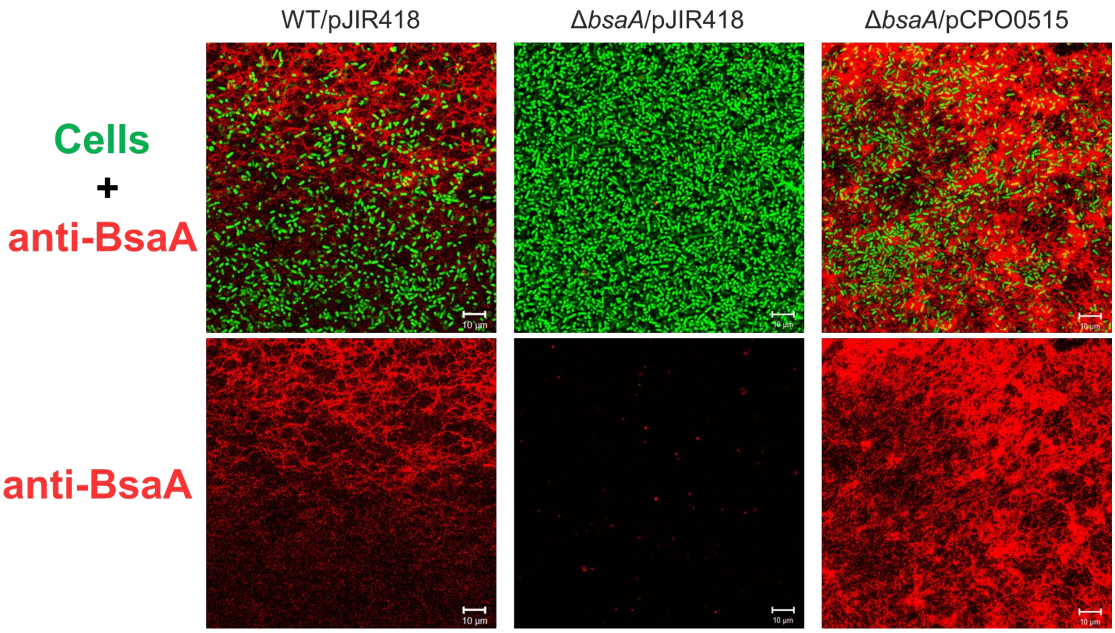

Supplementary Figure 8. Complementation of BsaA restores filamentous EPS production. Cells were anaerobically grown in GAM broth containing 2 mM lactose at 25°C for 2 days. The biofilms were fixed with 4% formaldehyde and then probed with anti-BsaA antibodies (red). Cells were stained with Syto9 (green). Filamentous anti-BsaA signals were detected in WT cells harboring pJIR418 (empty vector) and Δ*bsaA* cells harboring pCPO0515 (*bsaA* expression vector). We observed much amount of BsaA filamentous structure in Δ*bsaA* cells harboring pCPO0515 than WT harboring empty vector. The expression of *bsaA* in pCPO0515, which is under the control of the lactose-inducible *bgaL* promoter, would be higher than the native promoter of *bsaA* (*sipW* promoter). Scale bars are 10 μm.

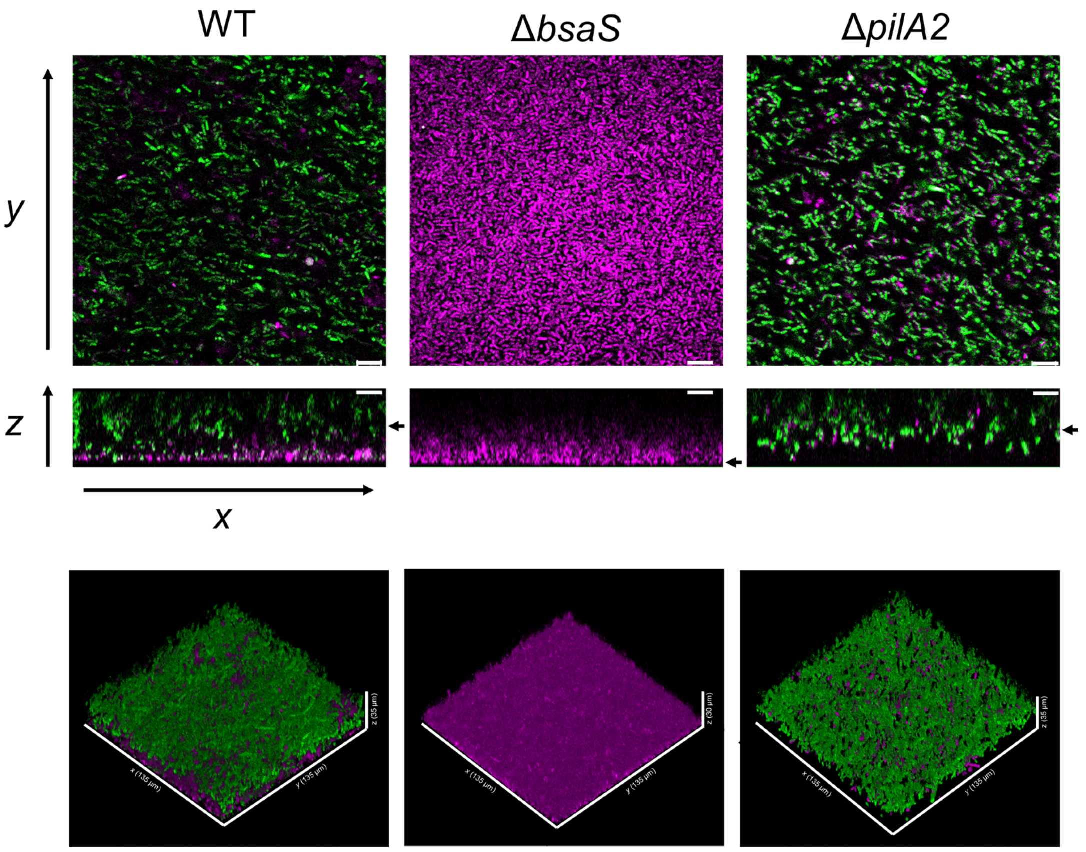

Supplementary Figure 9. Δ*bsaS* cells produce adhered biofilm at 25°C. *C. perfringens* cells harboring pCPE2005 (P_{sipW}-*evoglow-Pp1*) were anaerobically grown in GAM broth at 25°C for 24 h. Cells were stained with FM4-64 (magenta). We observed the biofilms using CLSM. Representative images of the *x-y* section and *x-z* section (top), and 3D image (bottom) of each biofilm are shown. We detected P_{sipW}-ON cells (green) in WT, and Δ*pilA2*, but not in Δ*bsaS*. WT and Δ*pilA2* form pellicle biofilms, whereas Δ*bsaS* forms adhered biofilms. Bar=10 μm.

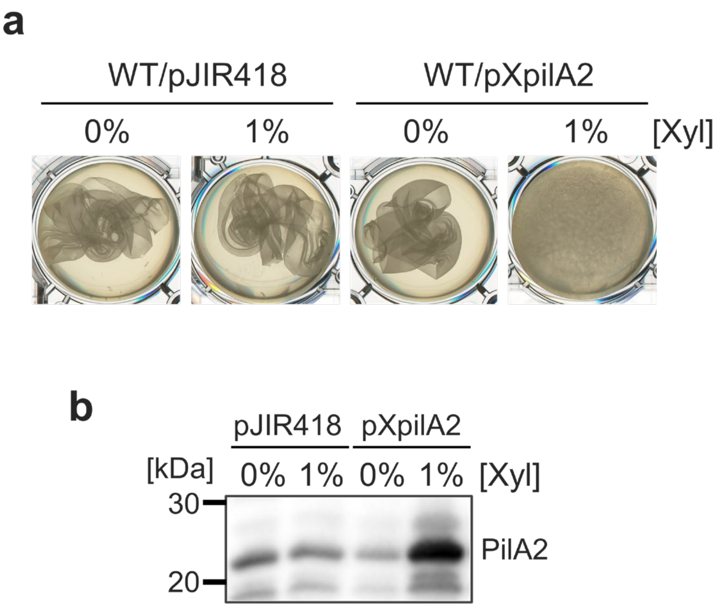

Supplementary Figure 10. Overproduction of PilA2 inhibits pellicle biofilm formation. (a) Pellicle biofilm formation of strains harboring plasmids expressing the *pilA2* gene. Cells were grown at 25°C for 2 days with or without xylose. The photographs show the pellicle biofilm after picking by gentle pipette aspiration. The transcription of the *pilA2* gene in pXpilA2 was under the control of the xylose-inducible promoter *xylB*. (b) Western blotting of PilA2 proteins. Whole cell proteins were extracted from the cultures described in Supplementary Figure 10a. The proteins were separated with 4-15% SDS-PAGE and probed with anti-PilA2 antibodies. PilA2 induced by 1% xylose inhibited pellicle biofilm formation.

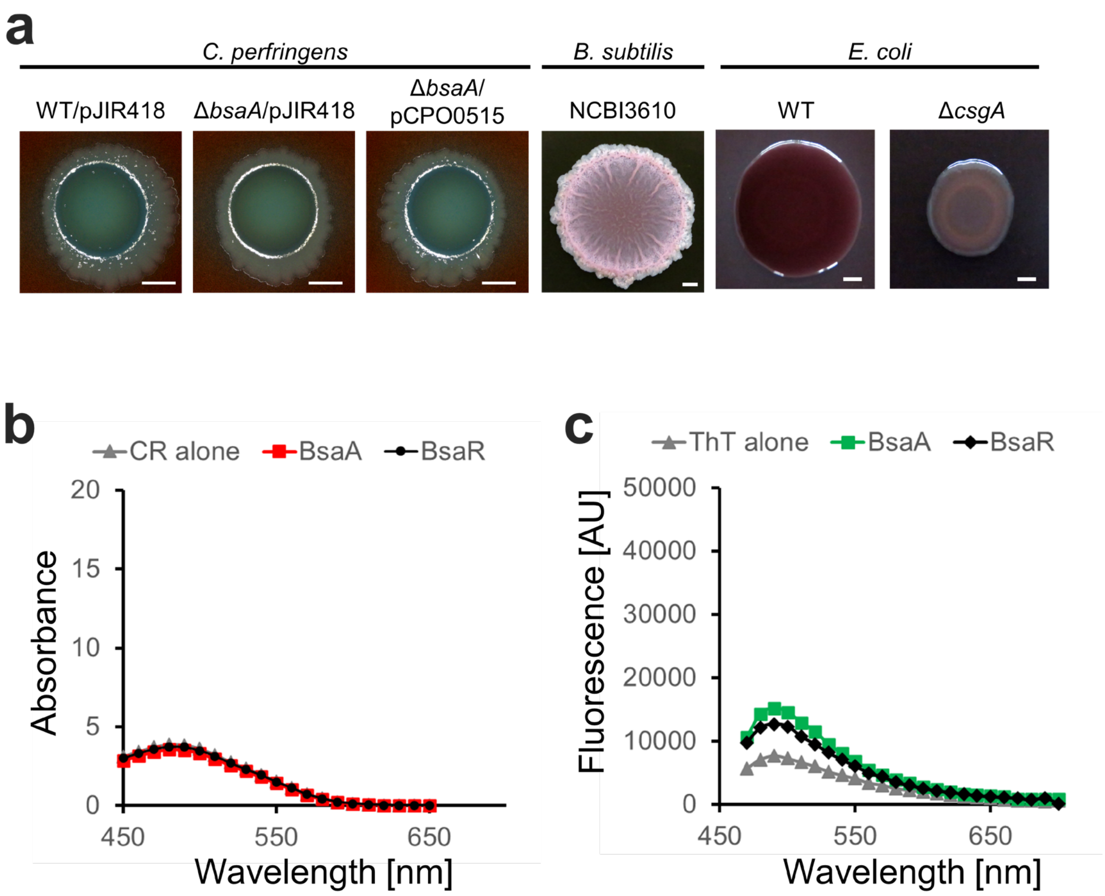

Supplementary Figure 11. BsaA proteins do not bind to Congo Red and thioflavin T. (a) Colony morphology on agar plates containing 40 μg/ml of Congo Red and 20 μg/ml of Coomassie Brilliant Blue G (CBB). *C. perfringens* were cultured in PGY medium. After overnight culture, we spotted 5 μl of the culture on GAM plates containing 1 mM lactose, Congo Red and CBB. *B. subtilis* and *E. coli* were grown in LB medium overnight. We spotted 5 μl of the culture on LBGM (1% tryptone, 0.5% yeast extract, 1% glycerol, 10 mM $MnSO_4$) or salt-free LB plates containing Congo Red and CBB. These plates were incubated at 25°C for 7 days (*C. perfringens*) or 4 days (*B. subtilis* and *E. coli*). Bar=0.5 mm. (b and c) Absorbance and fluorescence of BsaA proteins in the presence of Congo Red and Thioflavin T. Purified BsaA proteins (10 μg) were suspended in 100 mM NaCl and 20 mM Tris-HCl (pH 7.5) and mixed with 20 μM Congo Red (b) or Thioflavin T (c). The absorbance and the fluorescence (438-nm emission) of the suspension (100 μl) were measured using a microplate reader (Synergy H1, BioTek, Winooski, VT). Congo Red or Thioflavin T alone serve as negative controls.

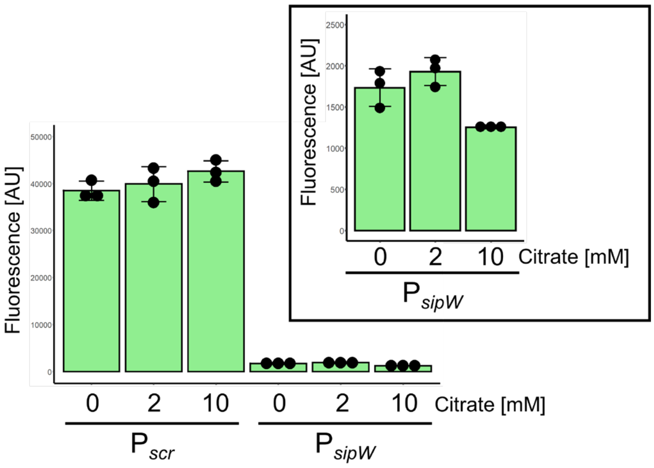

Supplementary Figure 12. Citrate does not influence P*sipW* expression. *C. perfringens* HN13 harboring pCPE2002 (P$_{scr}$-*evoglow-Pp1*) or pCPE2005 (P$_{sipW}$-*evoglow-Pp1*) were anaerobically grown in 1 ml of GAM containing citrate for 7 h at 37°C. The cells were washed with PBS and resuspended in 1 ml PBS. We measured the fluorescence (Ex/Em=450/495) of the suspension (100 µl) using a microplate reader. The means normalized by O.D.600 and standard deviations are represented.

Supplementary Table 1. Transposon mutants deficient in pellicle biofilm formation*

Locus (gene)	Number of mutants	Function
CPE0515 (*bsaA*)	2	Hypothetical protein
CPE0929	1	Glycerol dehydrogenase large subunit
CPE1512 (*reeS*)	1	Sensor histidine kinase ReeS
CPE1941 (*secF*)	1	Preprotein translocase subunit SecF
CPE1942 (*secD*)	1	Preprotein translocase subunit SecD
CPE2033 (*dnaK*)	1	Molecular chaperone DnaK
CPE2230	1	Serine protease Do
CPE2274	1	Nitrate extrusion protein NarK
CPE2418 (*nusG*)	1	Transcription antitermination protein NusG
CPE2507	2	Anaerobic ribonucleoside triphosphate reductase
rRNA/tRNA	23	Ribosomal RNA/transfer RNA

*Amongst 51 mutants, 9 mutants were not tested, and 7 mutants could not read. Thirty-five mutants successfully sequenced are listed.

Supplementary Table 2. Strains and plasmids used in this study

Strain	Genotype or relevant characteristics	Source or reference
C. perfringens		
13	Wild type	Shimizu *et al.* (2002)
HN13	*galK*, *galT* in-frame deletion mutant of 13	Nariya *et al.* (2011)
NO25	*pilA2* in-frame deletion mutant of HN13	Obana *et al* (2014)
NO41	*sipW*, *bsaA* in-frame deletion mutant of HN13	In this study
NO42	*bsaA* in-frame deletion mutant of HN13	In this study
NO43	*bsaR* in-frame deletion mutant of HN13	In this study
NO44	*bsaS* in-frame deletion mutant of HN13	In this study
NO45	*bsaR*, *bsaS* in-frame deletion mutant of HN13	In this study
NO46	*bsaB* in-frame deletion mutant of HN13	In this study
NO47	*bsaC* in-frame deletion mutant of HN13	In this study
NO48	*srtB* in-frame deletion mutant of HN13	In this study
NO77	*bsaD* in-frame deletion mutant of HN13	In this study
E. coli		
DH5α	Used for cloning	TAKARA
M15/pREP4	Used for protein expression	Qiagen
**Plasmid**		
pJIR418	*E. coli-C. perfringens* shuttle vector, Cm^R, Em^R	Sloan J *et al.* (1992)
pCPO0514FLAG	FLAG-tagged *sipW* expression under control of lactose inducible promoter (*bgaL*)	In this study
pCPO0515	*bsaA* expression under control of lactose inducible promoter (*bgaL*)	In this study
pCPE2001	*colA*-processed 5'UTR-evoglow-Pp1-Cp-flag in pJIR418	In this study
pCPE2002	P_{scr}-*colA*-processed 5'UTR-*evoglow-Pp1-Cp-flag* in pJIR418	In this study
pCPE2005	P_{sipW}-*colA*-processed 5'UTR-*evoglow-Pp1-Cp-flag* in pJIR418	In this study
pXCH	Xylose-inducible promoter-harboring plasmid	
pXCH-sipW	*sipW*-carrying pXCH	In this study
pXCH-sipW-bsaA	*sipW-bsaA*-carrying pXCH	In this study
pXCH-pilA2	*pilA2*-carrying pXCH	In this study
pQE60	IPTG-inducible C-terminal His6 added protein expression vector	Qiagen
pQE60-BsaR	BsaR-His6 expression vector	In this study
pQE60-BsaA	BsaA-His6 expression vector	In this study

Supplementary Table 3. Oligonucleotides used in this study

Primer ID	Sequence	Use
NOB-0777	ggccgtcgacctatgccatctcttttaaatagtg	*sipW* mutant construction
NOB-0879	AAAGTGATTCTGGAGGAATAAGAAAGAAAAGTTAG	*sipW* mutant construction
NOB-0880	TTTCTTTCTTATTCCTCCAGAATCACTTTTCTTAG	*sipW* mutant construction
NOB-0881	GCGCggatccCTTTCAAGCTTATCTCCTGG	*sipW* mutant construction
NOB-0786	ggccgtcgacctttctgcatcctgcttatg	*bsaA* mutant construction
NOB-0787	gtacctgcacctaacaatttaaattacctcccagt	*bsaA* mutant construction
NOB-0788	aggtaatttaaattgttaggtgcaggtactaatgc	*bsaA* mutant construction
NOB-0789	gcgcggatccccatcttcatctggctcttg	*bsaA* mutant construction
NOB-0818	GGCCgtcgacACAACTCATAGAGCTATAGA	*bsaB* mutant construction
NOB-0819	cccacctaactaCTTCATCCTTTTCACTCCTTGCA	*bsaB* mutant construction
NOB-0820	aaggATGAAgtAGttaggtggggagagatATGAAG	*bsaB* mutant construction
NOB-0821	GCGCggatccCTTGACCAAATCTTGAATCA	*bsaB* mutant construction
NOB-0822	GGCCgtcgacATCCAGATGGACAAACTGCA	*bsaC* mutant construction
NOB-0823	atcctttattgaCTTCATATCTCTCCCCACCTAAC	*bsaC* mutant construction
NOB-0824	gagatATGAAgtCAATAAAGGATGAAAAATACTTC	*bsaC* mutant construction
NOB-0825	GCGCggatccTATAAACTCTTCCAACAGGTA	*bsaC* mutant construction
NOB-0801	GGCCgtcgacAACAGATGTTGTTTCTAAGG	*bsaR* mutant construction
NOB-0802	ATTACATATCGCcaTCGCATATTGCTATATTTAAC	*bsaR* mutant construction
NOB-0803	GCAATATGCGAtgGCGATATGTAAttttATGTATG	*bsaR* mutant construction
NOB-0804	GCGCggatccCACAAGCTTCTATAGCATTATC	*bsaR* mutant construction
NOB-0805	GGCCgtcgacGTTATAAATAGTTCAATAAAGGATG	*bsaS* mutant construction
NOB-0806	TATGCACTGccCGTACATACATAAAATTACATATC	*bsaS* mutant construction
NOB-0807	TTTATGTATGTACGggCAGTGCATAAAAATCATGG	*bsaS* mutant construction
NOB-0808	ATTTGGATATATTCCACCCTTAGTTG	*bsaS* mutant construction
NOB-0826	GGCCgtcgacCAGCATCCTCCTTATTGAAATC	*srtB* mutant construction
NOB-0827	CTATTCTAGCCATaatactcacctctaaataacga	*srtB* mutant construction
NOB-0828	gaggtgagtattATGGCTAGAATAGTAGTGGTAGC	*srtB* mutant construction
NOB-0829	GCGCggatccTGGGATATGAATAGGAATATGACAG	*srtB* mutant construction
NOB-1196	GGCCgtcgacAACTAAACTGGTATTTGCAATTGGA	*bsaD* mutant construction
NOB-1197	TAGCTTCTATTAAAGATCCATTCTTCATatttttcctcca	*bsaD* mutant construction
NOB-1198	aatATGAAGAATGGATCTTTAATAGAAGCTAATATATCAG	*bsaD* mutant construction
NOB-1199	GCGCggatccgatagatttaaatttcaaaagatcc	*bsaD* mutant construction
NOB-0488	GGCCGAGCTCAAGTCTAATTAAGACTTTAG	*bgaR*-P*bgaL* cloning
NOB-0489	GCGCGGATCCCATTTTACCCTCCCAATACA	*bgaR*-P*bgaL* cloning
NOB-0921	GCCggatccAGTAAGAAGAAAATAATAGGCTTATG	pCPO0515 construction
NOB-0903	GCGCgtcgactttcactccttgcatcatgg	pCPO0515 construction
NOB-0490	GGCCagatctATGAAAAAAGGTATAAAAAT	pCPO0514-FLAG construction
NOB-0905	GCGCgtcgacACTTTTCTTTCTTATTCCTAAATA	pCPO0514-FLAG construction
NOB-0455	GGCCgtcgacGATTATAAAGATGATGACGATAAAGGTTAAAAA TTTTAATTAGGATGGAG	FLAG tag and *lipA* intrinsic terminator amplification
NOB-0456	GCGCaagcttTAAGCTATAGTATAAACAAG	*lipA* intrinsic terminator amplification
NOB-0932	GAAGAAGTTTTGCATTTATTAACTTCAGTTTTGTTAGCTC	pCPE2001 construction
NOB-0933	GCTAACAAAACTGAAGTTAATAAATGCAAAACTTCTTCAG	pCPE2001 construction
NOB-0600	GGCCtctagaGTATATAAGAAAACTTCAGC	pCPE2001 construction
NOB-0929	GGCCgtcgacTTAATGTTTTGCTTGTCCTTG	pCPE2001 construction
NOB-0916	GGCCggatccgctttgatatagctcatttttaagtg	pCPE2002 construction
NOB-0915	GCGCtctagactttttcattctaacatataagtc	pCPE2002 construction
NOB-0780	ggccggatccctacgataatttcaatattacacc	pCPE2005 construction
NOB-0914	GCGCtctagacagattatttataacatcttaagta	pCPE2005 construction
NOB-0981	GGCCgtcgacAAAAAAGGTATAAAAATTTTTTATAATATTTTAT TTTATGG	pXCH-sipW and pXCH-sipW-bsaA construction

NOB-0982	GGCCagatctCTAACTTTTCTTTCTTATTCCTA	pXCH-sipW construction
NOB-0983	GGCCagatctTTATTTATGTGCATTAGTACCTGC	pXCH-sipW-bsaA construction
NOB-1086	CATatgcatccctccttgaatg	pXCH-pilA2 construction
NOB-1087	TCTAGATCTCATCATCATCATCATC	pXCH-pilA2 construction
NOB-1090	ggagggatgcatATGAATACAAAAAAACAAAACAAAAAAAAG	pXCH-pilA2 construction
NOB-1091	atgatgagatctagaCTATTGATTATTTCTTTCATTAGTTAC	pXCH-pilA2 construction
NOB-0798	TTAACCATGGTTTTTACATCAAGTGATTCTG	pQE60-bsaA construction
NOB-0799	ATCTGGATCCTTTATGTGCATTAGTACCTGC	pQE60-bsaA construction
NOB-0910	GGCCccatggTAAATATAGCAATATGCGATGATGA	pQE60-bsaR construction
NOB-0911	GCGCggatccCATATCGCCTATTCTATTCATTAAG	pQE60-bsaR construction
NOB-0538	GAAAGGGTTCACGCTAATTG	DIG-labeled *pilA2* probe
NOB-0539	ACTGATGCTGATGTGTTTGA	DIG-labeled *pilA2* probe
NOB-0794	GAAAATAATAGGCTTATGTATAGCC	DIG-labeled *bsaA* probe
NOB-0795	CATTTACTGCACCATTAGCAGCTTG	DIG-labeled *bsaA* probe
NOB-0127	GTTTTTTAACTATACTTATAGCATTTTTAC	DIG-labeled *sipW* probe
NOB-0128	CTTCTTACTCAATTTAAATTACCTCCCAG	DIG-labeled *sipW* probe
NOB-0129	GCAATATGCGATGATGAAAAAGTCCAGCG	DIG-labeled *bsaR* probe
NOB-0130	CTTCTTCTAATGTTGCTATTTTCATATTG	DIG-labeled *bsaR* probe
NOB-0482	CGCGGAATTCTGCGTTTGCTGGCTTTGATG	5' RLM-RACE
NOB-0483	GCTGATGGCGATGAATGAACACTG	5' RLM-RACE
NOB-0494	GCGCggatccTTCCTGGCTTTATAGTAGGA	5' RLM-RACE
NOB-0591	AAGGGAATTCTTGTAATTAAGAAGGAGTGA	pMOD-2-ermBP construction
NOB-0592	AAGGAAGCTTTTTACAAAAGCGACTCATAG	pMOD-2-ermBP construction
NOB-0301	TGTTCAAGAAGTTATTAAGTCGGGAGTGC	Sequencing of transposon mutants
NOB-0302	CTTCTTTTACGTTTCCGGGTACAATTCG	Sequencing of transposon mutants

Original blots

Figure 1C

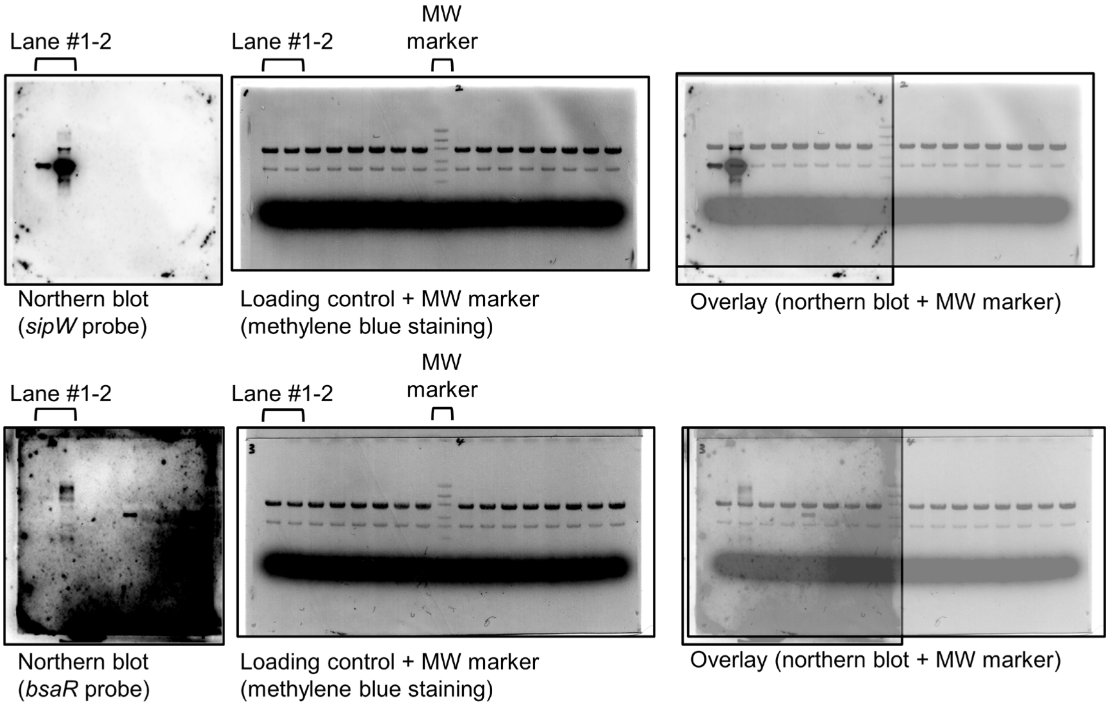

Figure 2A

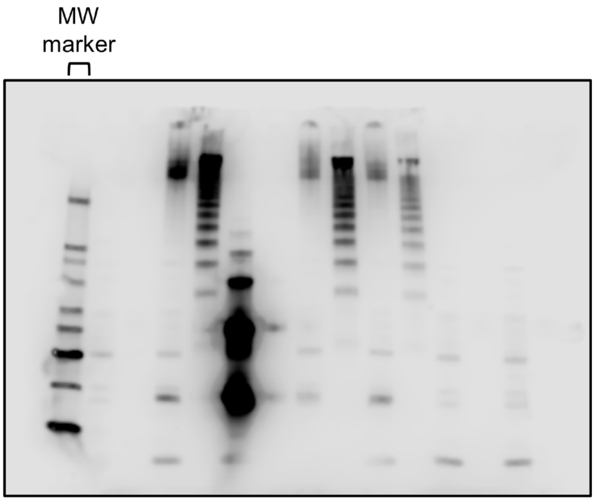

Western blot (anti-BsaA)

Figure 2B

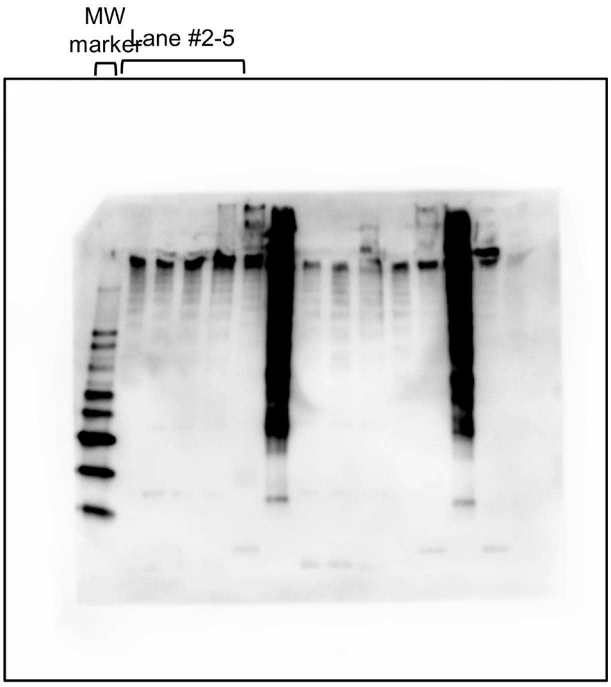

Western blot (anti-BsaA)

Figure 3A

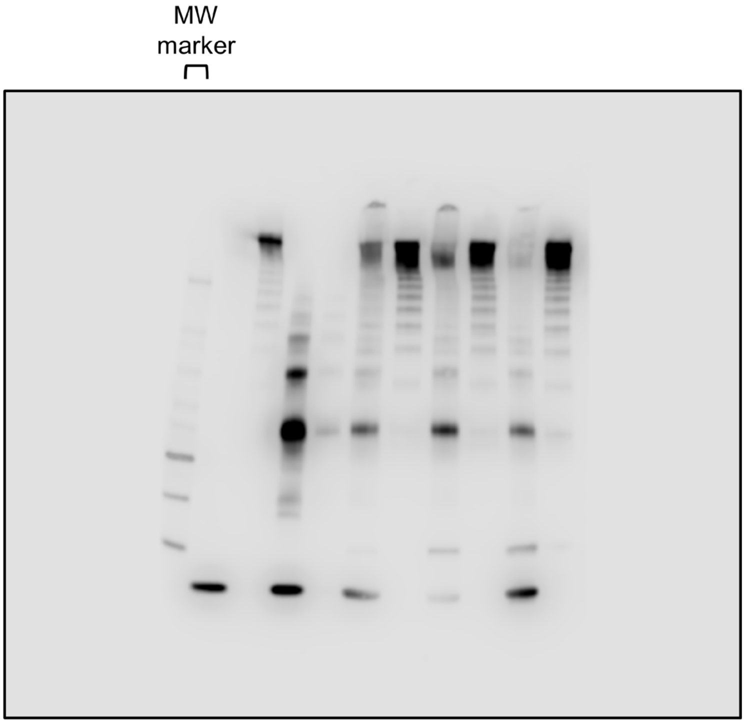

Western blot (Anti-BsaA)

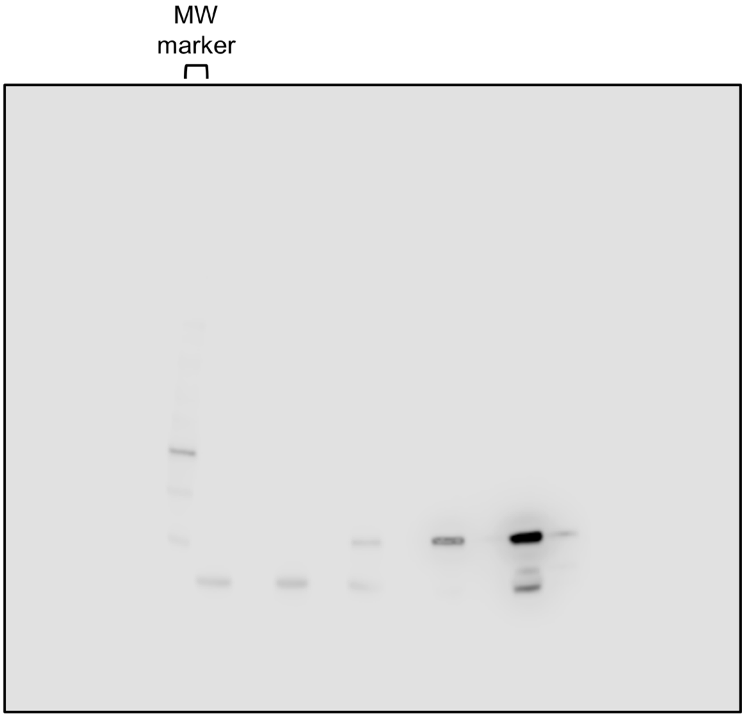

Western blot (Anti-FLAG)

Figure 8C

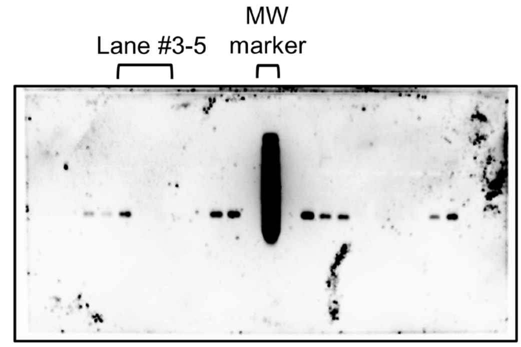

Northern blot (*pilA2*)

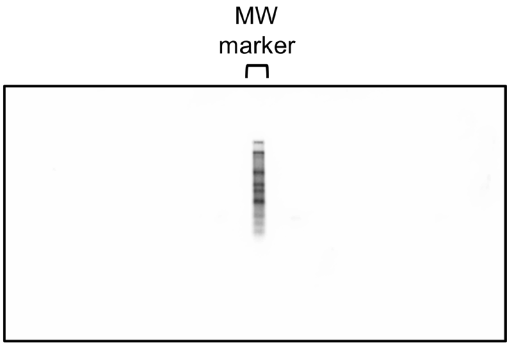

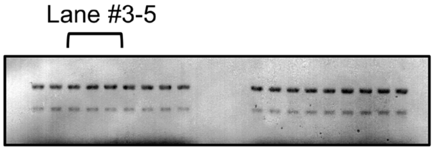

Loading control (methylene blue staining)

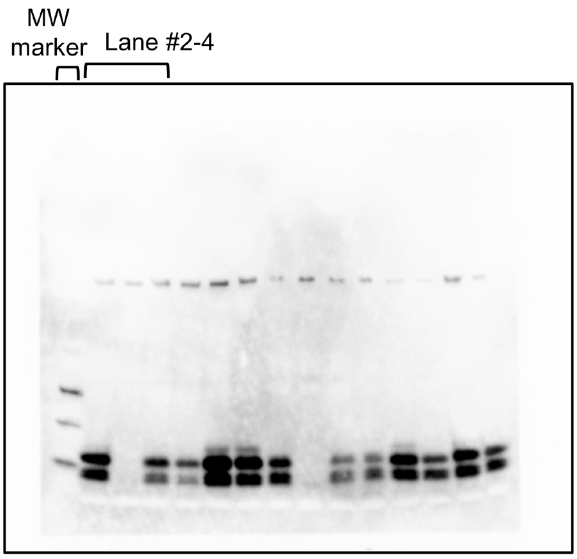

Western blot (Anti-PilA2)

Supplementary Figure 1B

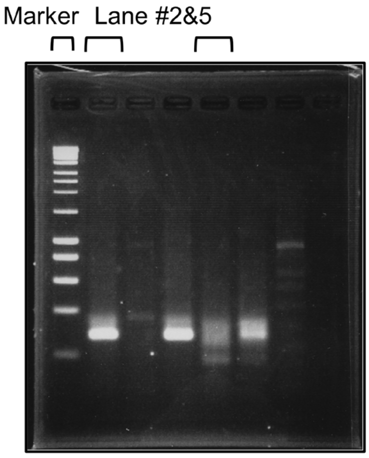

Agarose gel stained with ethidium bromide

Supplementary Figure 2B

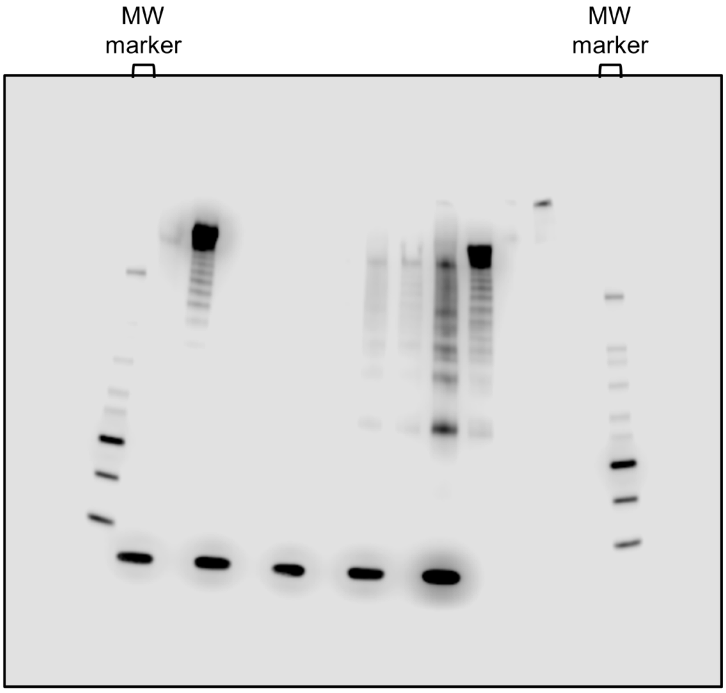

Western blot (Anti-BsaA)

Supplementary Figure 4B

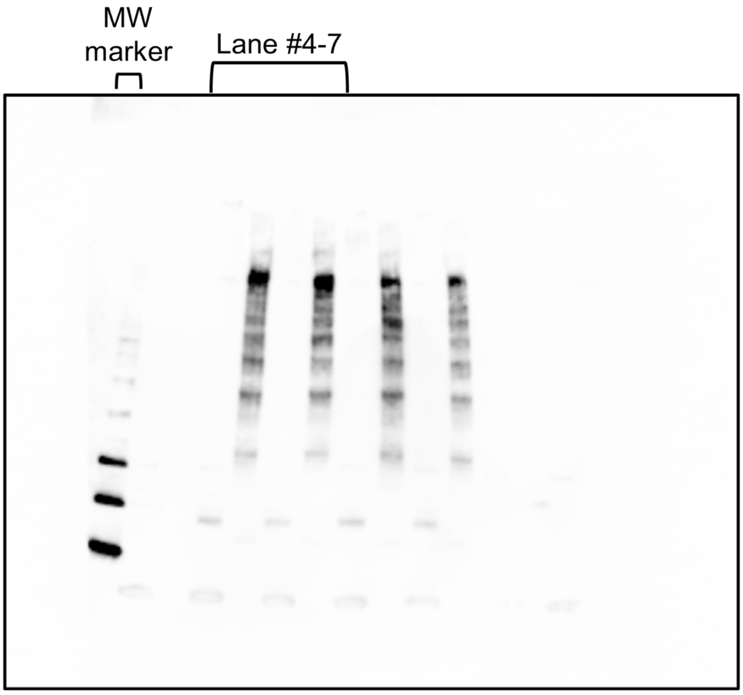

Western blot (Anti-BsaA)

Supplementary Figure 5C

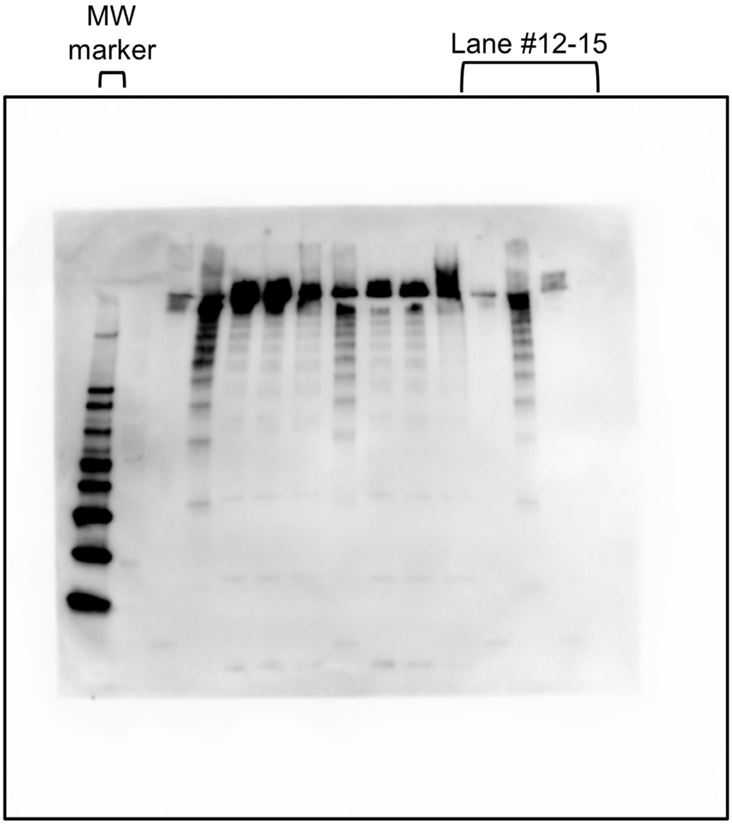

Western blot (Anti-BsaA)

Supplementary Figure 8A

Lane #3&8, 9

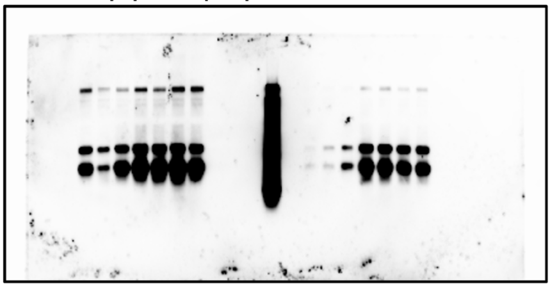

Northern blot (*sipW-bsaA* probe)

MW marker

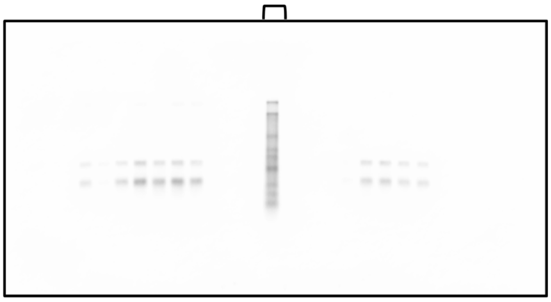

MW marker

Lane #3&8, 9

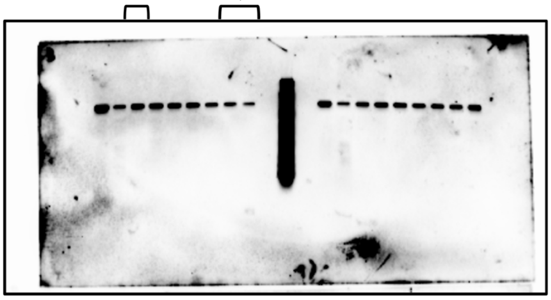

Northern blot (*colA* probe)

MW marker

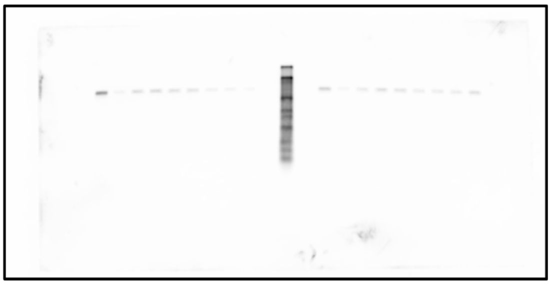

MW marker

Supplementary Figure 8B, C, D

Lane #1-4 P_{sipW} | Lane #7-10 P_{abrB} | Lane #13-14 P_{scr}

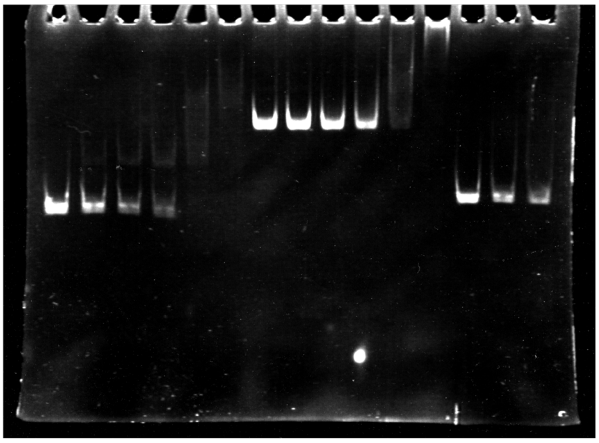

Acrylamide gel stained with ethidium bromide

Supplementary Figure 10B

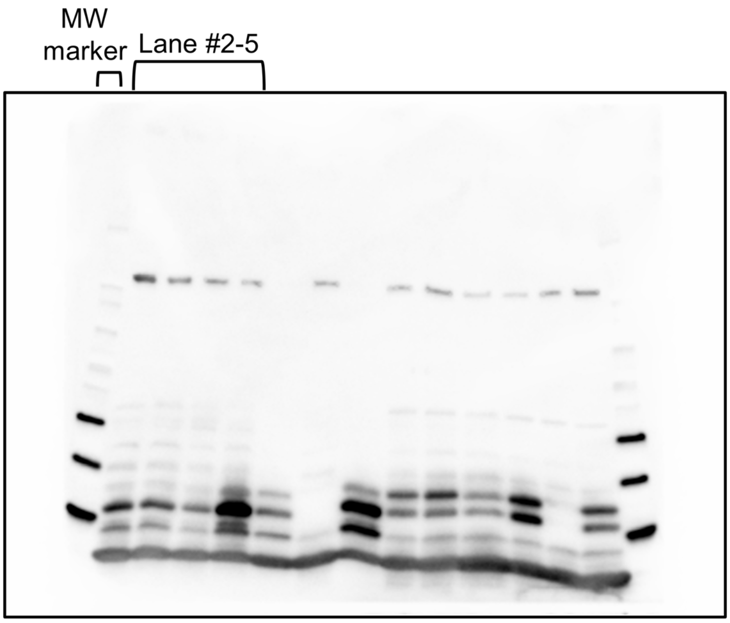

Western blot (Anti-PilA2)
